# Effects of heteroatom doping on hydrogen uptake in tungsten oxide

**DOI:** 10.1039/d5sc08564k

**Published:** 2026-01-15

**Authors:** Noah P. Holzapfel, Nikolaos Effraim Papamatthaiakis, Jay R. Paudel, Giannis Mpourmpakis, Ethan J. Crumlin, Veronica Augustyn

**Affiliations:** a Department of Materials Science and Engineering, North Carolina State University Raleigh NC 27695 USA vaugust@ncsu.edu; b School of Chemical Engineering, National Technical University of Athens (NTUA) Athens GR-15780 Greece; c Chemical Sciences Division, Lawrence Berkeley National Laboratory Berkeley CA 94720 USA; d Department of Chemical and Petroleum Engineering, University of Pittsburgh Pittsburgh PA 15261 USA; e Advanced Light Source, Lawrence Berkeley National Laboratory Berkeley CA 94720 USA

## Abstract

Redox-active transition metal oxides (TMOs) that can undergo proton-insertion coupled electron transfer (PICET) are promising candidates for catalyzing molecular conversion reactions which require the transfer of hydrogen atoms (or the thermochemical equivalent, H^+^, e^−^). Herein, we studied the effects of isovalent (Mo^6+^) and aliovalent (V^5+^ and Nb^5+^) heteroatom doping on the electrochemical PICET behavior of monoclinic tungsten oxide (WO_3_). Cyclic voltammetry in aqueous acidic electrolytes shows that the addition of redox-active heteroatoms (Mo^6+^ and V^5+^) leads to systematic shifts in redox couple half-wave potentials (*E*_1/2_), broadening, and an overall decrease in the current response. Conversely, the non-redox active heteroatom (Nb^5+^) only reduces the current response with no observable peak-current broadening. This broadening is attributed to changes in the proton binding affinities of oxygen in different chemical environments, *i.e.*, bridging different pairs of redox-active transition metal cations. We determined the hydrogen bond dissociation free energy (H BDFE) values to elucidate the thermodynamic effect of heteroatom substitution. Density functional theory calculations reveal a differentiation in the hydrogen binding and oxygen vacancy formation energies between heteroatom doped structures. The PICET-induced structural phase transitions of the pristine and doped samples were further probed with *operando* electrochemical X-ray diffraction (EC-XRD) and with *ex situ* chemical reduction. The broadening of the potential-dependent current response with increased heteroatom doping manifests in the *operando* EC-XRD results as prolonged structural regions where multiple hydrogen bronze phases exist and the appearance of cubic bronze phases at lower degrees of reduction compared to pristine WO_3_.

## Abstract 2

Introduction of heteroatom dopants into WO_3_ increases the distribution of oxygen chemical environments, thus tuning hydrogen binding energies and broadening the current associated with proton-insertion coupled electron transfer.

## Graphical abstract

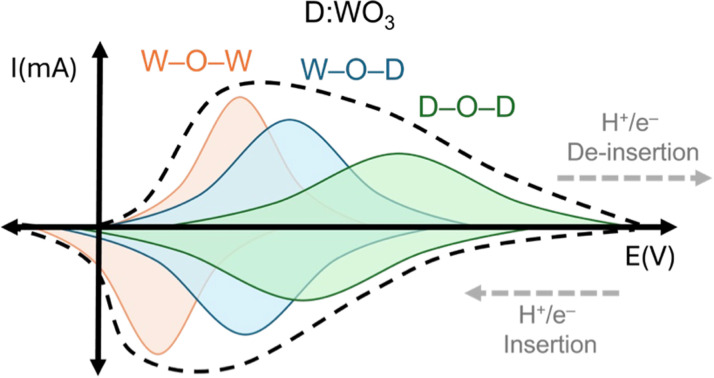

## 1 Introduction

Early transition metal oxides (TMOs), such as those based on titanium^1,2^ and tungsten,^3,4^ can take up hydrogen atom equivalents (*i.e.*, proton/electron pairs) *via* proton-insertion coupled electron transfer (PICET) reactions from acidic environments.^5^ This property can be utilized for a variety of electrochemical and catalytic applications, including in aqueous energy storage,^5–10^ water electrolysis,^4,11^ and heterogeneous hydrogen transfer catalysis.^12,13^ All of these require the transfer of protons and electrons across a phase boundary (*i.e.*, electrochemical interface, TMO/liquid, or TMO/gas). In TMOs, the thermochemical properties associated with PICET can be tuned across a variety of length scales, from the chemical composition and crystal structure to defect concentration, particle morphology, and particle size.^2,14,15^ This high degree of tunability enables various strategies for controlling proton-coupled electron transfer (PCET) reaction kinetics and selectivity.

**Fig. 1:**
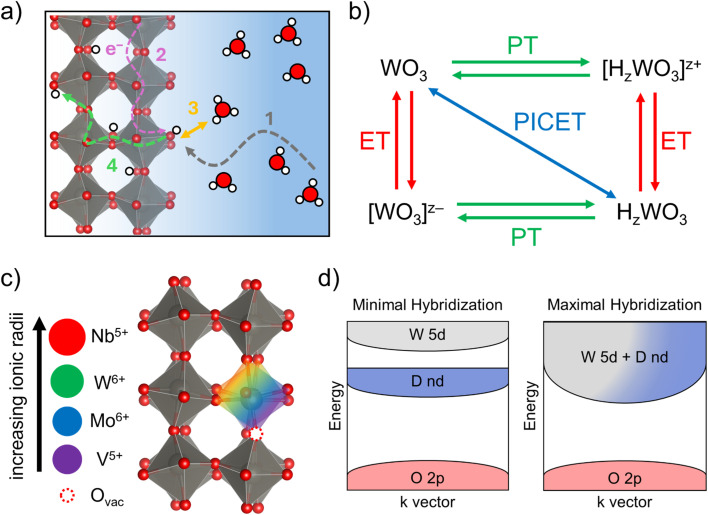
Fundamental steps of PICET (a) and reaction square scheme for PICET into WO_3_ (b). Visual representations of heteroatom doping into WO_3_ with a qualitative comparison of the ionic radii for the heteroatom dopants explored here (c) and projected electronic structures (d) of the WO_3_ host upon heteroatom doping.

At the atomic level, reversible PICET involves the formation and homolytic cleavage of covalently bonded hydrogen atoms within the host structure. In TMOs, the inserted protons are localized to the oxide ligands, forming hydroxyl (M–OH) groups while the electrons populate conduction band states associated with the transition metal cations.^5^ The fundamental steps of PICET involve (1) mass transport of protons in the liquid electrolyte; (2) transfer of an electron to the solid host; (3) proton-coupled electron transfer (PCET) at the interface; and (4) mass transport of the proton in the solid state (Fig. 1a). The interfacial PCET reaction may proceed sequentially or in a concerted fashion and is represented by a reaction square scheme, as shown in Fig. 1b, using the example of PICET into WO_3_. The energy change associated with forming the O–H bond, which requires both PT and ET steps, is represented as the diagonal reaction within the square scheme. Also termed the bond dissociation free energy (BDFE), this parameter can be quantified from the pH-dependence of the PICET half-wave potential (*E*_1/2_).^16^ By understanding the factors which govern such PICET reactions on TMOs, we can better design materials for energy and catalytic applications.

Matson *et al.* showed that heteroatom doping of polyoxotungstate clusters, which are molecular-scale analogs of extended tungsten oxides, allows for control over the PCET thermochemistry.^17–20^ For example, the Lindqvist-type cluster, (TBA)_2_[W_6_O_19_], undergoes two ET-reactions, with no measurable dependence on the electrolyte p*K*_a_, suggesting that the reduction is not coupled to proton transfer (PT). Vanadium doping of this cluster to form (TBA)_3_[VW_5_O_19_] led to a change in the redox behavior, wherein the two ET-reactions of (TBA)_2_[W_6_O_19_] gave way to a single redox-couple in (TBA)_3_[VW_5_O_19_] associated with PCET at the V-site.^20^ The Keggin-type cluster, (TBA)_3_[PW_12_O_40_], undergoes four PCET reactions and the BDFEs could be increased by >20 kcal mol^−1^ by substituting a single W-site for V.^19^ The complete substitution of W^6+^ with Mo^6+^, forming the isostructural Keggin-type polyoxomolybdate cluster, (TBA)_3_[PMo_12_O_40_], was also found to increase the BDFE by 20 kcal mol^−1^.^18^ These molecular studies emphasize the impact of heteroatom doping and the importance of transition metal identity, dopant location, and orbital hybridization on PCET, serving as an inspiration to investigate the role of heteroatom dopants on PICET in WO_3_.

In extended TMOs, the incorporation of heteroatom dopants can tune the electronic structure by changing the distribution of the electron density near the Fermi level. This effect can be achieved through the introduction of either isovalent or aliovalent transition metal dopants. The ability (or lack thereof) for the dopant ion orbital states to hybridize with the oxide p-orbitals perturbs the relative energies of conduction band states, changing the energetic requirements for electron transfer and delocalization.^21^ This compositional tuning not only modifies the electronic energy landscape of the TMO but can also impact PICET. Both isovalent and aliovalent doping can alter metal–oxygen bond lengths and angles, which in turn can modify the stable proton insertion sites. Miu *et al.* reported that PICET into WO_3_ could be accurately predicted by analyzing the acid–base properties of the oxide host, further demonstrating a pair-wise correlation between hydrogen binding, Bader charges, and electronic density of states band centers, which were used as descriptors for the oxide Lewis acidity/basicity.^22^ Unique to aliovalent doping is the possibility for secondary compositional changes, such as the formation of vacancies or interstitial ion inclusions needed to compensate for the change in oxidation state (Fig. 1c), which could further alter the proton insertion behavior. Considering this, we propose two extreme hypothetical scenarios to describe the influence of heteroatom doping on PICET in TMOs: (1) limited hybridization between the dopant and host results in discreet PICET reactions such as what is seen in polyoxometalate clusters or (2) extensive hybridization induces systemic changes in the PICET reactions associated with the host transition metal (Fig. 1d). Coupled with these atomic-scale changes is the possibility of heteroatom doping to alter particle size or morphology, which could dramatically affect the quantity or availability of reactive surface sites to participate in PCET.

Here, we investigate how isovalent (Mo^6+^) and aliovalent (V^5+^ and Nb^5+^) heteroatom doping affects the thermodynamics and kinetics of proton-insertion coupled electron transfer (PICET) in monoclinic (*γ*) WO_3_. Structural changes upon doping were characterized by XRD and Raman spectroscopy, while SEM and BET analyses assessed the microstructure and surface area. The distribution of the heteroatoms within the sample particles was assessed with depth-dependent XPS measurements. Electrochemical PICET behavior was studied using cyclic voltammetry at varying scan rates and pH in acidic aqueous electrolytes. *Operando* XRD and *ex situ* chemical reduction were used to probe the PICET-induced structural dynamics. Redox-active dopants (Mo^6+^ and V^5+^) shifted the onset of structural transitions, whereas the non-redox-active dopant (Nb^5+^) suppressed PICET and increased the hydrogen evolution onset potential. DFT calculations were further used to calculate the hydrogen binding energies (HBEs), oxygen vacancy formation energies (OVFEs), and net charge of the metal atoms within the doped systems. The HBEs were found to be dependent on the identity of the transition metals which coordinate to the proton accepting oxygen. These findings demonstrate that heteroatom doping provides a tunable handle for modulating hydrogen bonding and PCET thermochemistry in transition metal oxides.

## 2 Experimental methods

### 2.1 Chemicals

All chemicals were used as received. Na_2_WO_4_·2H_2_O (99+%), concentrated HCl (12.1 M, Certified ACS Plus), Na_2_MoO_4_·2H_2_O (99+%), concentrated HNO_3_ (15.6 M, Certified ACS), NaVO_3_ (96%), NbCl_5_ (99.9%), TEMPO (98+%), toluene (Certified ACS), acetylene black (99.9+%), acetone (ACS Certified), ethanol (anhydrous, histological grade), concentrated H_2_SO_4_ (18 M, Certified ACS Plus), and NaOH (Certified ACS) were purchased from ThermoFisher. H_2_PtCl_6_ (8% by weight in H_2_O), H_3_PO_2_ (50% by weight in H_2_O), lithium (99.9% trace metals basis), 1 M LiPF_6_ in ethylene carbonate (EC) : dimethyl carbonate (DMC) (50 : 50 battery grade), and n-methyl pyrrolidone (NMP, anhydrous, 99.5%) were purchased from Millipore Sigma. Poly(vinylidene fluoride) (PVDF; Kynar HSV 900) was purchased from Arkema.

### 2.2 Materials synthesis

Monoclinic tungsten oxide dihydrate (WO_3_·2H_2_O) was synthesized using the acid precipitation method developed by Freedman.^23^ 50 mL of a 1 M Na_2_WO_4_·2H_2_O aqueous solution was added dropwise to 450 mL of a 3 M HCl solution that was stirred at 300 rpm at room temperature. This mixture was allowed to stir for three hours, and the precipitate was collected *via* vacuum filtration and washed with DI water until the pH of the rinse solution reached ∼6. Monoclinic tungsten trioxide (γ-WO_3_) was formed by thermally dehydrating the as-prepared WO_3_·2H_2_O at 300 °C for 5 hours in air in a box furnace.

The heteroatom doped samples (Mo^6+^, V^5+^, and Nb^5+^:WO_3_) were prepared by modified acid-precipitation methods. For the Mo-containing compounds, first a fixed amount of Na_2_WO_4_·2H_2_O was added to a 20 mL scintillation vial with a magnetic stir bar. Next, a stoichiometric amount of Na_2_MoO_4_·2H_2_O was added to the vial along with 2.5 mL of DI H_2_O. The precursor masses and solvent volumes used for the Mo-doped sample syntheses are given in Table S1. The sample was allowed to stir until all the solids were completely dissolved. This solution was then added dropwise to a separate vial containing 10 mL of a 6 M HNO_3_ solution in DI H_2_O. Upon addition, a solid precipitate formed. The sample was allowed to continue stirring for three hours to promote crystallinity and ensure reaction completeness. The powder product was then separated *via* vacuum filtration, washed multiple times with water (pH of rinse solution ∼6) and once with acetone, and then dried at 60 °C in air. The powder was then ground for 10 minutes with an agate mortar and pestle, then loaded into an alumina crucible and annealed in air at 300 °C for 5 hours in a box furnace.

For the V-containing compounds, it was found that excess precursor reagent, NaVO_3_, was required to promote V-incorporation into the WO_3_ host phase. The NaVO_3_ and Na_2_WO_4_·2H_2_O precursors were dissolved in 5 mL of DI H_2_O in a 20 mL scintillation vial under constant heating and stirring at 80 °C in an oil bath. Once fully dissolved, the solution was allowed to cool to room temperature before being pipetted into a separate vial containing 10 mL of a 6 M HNO_3_ solution in DI water. Upon addition, a solid precipitate formed. The sample was allowed to continue stirring for three hours to promote crystallinity and ensure reaction completeness. The powders were separated and annealed following the same procedure as that for the Mo-containing samples. Precursor masses and solvent volumes used for the V-doped sample syntheses are also given in Table S1.

For the Nb-containing compounds, NbCl_5_ was weighed separately and added into a 20 mL scintillation vial inside a nitrogen-filled glove box (MBraun, O_2_ < 0.5 ppm, H_2_O < 0.5 ppm). The vial was capped and removed from the glovebox and the powder was subsequently dissolved in 1 mL of ethanol. Separately, Na_2_WO_4_·2H_2_O was dissolved in 2 mL of DI H_2_O. Once both solids were fully dissolved in their respective solvents, the solutions were pipetted simultaneously into a separate vial containing 10 mL of a 6 M HCl solution in DI H_2_O. HCl was used instead of HNO_3_ for safety considerations associated with the reaction of HNO_3_ with ethanol. Upon addition, a solid precipitate formed. The sample was allowed to continue stirring for three hours to promote crystallinity and ensure reaction completeness. The powders were separated and annealed following the same procedure as that for the Mo-containing samples. A table of precursor masses and solvent volumes used for the Nb-doped sample syntheses are also given in Table S1.

### 2.3 Physical characterization

Powder X-ray diffraction (PXRD) data were collected on a PANalytical X'Pert Pro powder diffractometer (45 kV, 40 mA, sealed Cu X-ray tube, Kα_1_, Kα_2_*λ* = 1.5406 Å, 1.5444 Å) equipped with an X'Celerator position sensitive detector in Bragg–Brentano geometry. Pawley refinements of the XRD data were performed using Topas Academic (V7).^24^ Crystal structure images were generated using Vesta 3.^25^ For direct comparison with synchrotron diffraction measurements all angular ranges were converted to the scattering vector, *Q*, in reciprocal space (Å^−1^) using the equation *Q* = (4π/*λ*) × sin (2*θ*/2). X-ray fluorescence (XRF) spectroscopy was performed on a Rigaku SuperMini200 sequential benchtop WDXRF spectrometer. For XRF measurements, approximately 1 g of powdered sample was enclosed within a Mylar film pouch before loading into the sample holder. The XRF results were corrected for elemental results associated with the Mylar film. Raman spectroscopy was performed using a WiTEC alpha 300R confocal Raman spectrometer with a laser wavelength of 532 nm and a 100× objective lens. Scanning electron microscopy (SEM) was performed on a Hitachi SU8700 equipped with an Everhart–Thornley detector. Samples were measured with an accelerating voltage of 2 keV with no applied stage bias. Brunauer–Emmett–Teller (BET) N_2_ sorption isotherms were obtained on a TriStar Micrometrics setup at 77 K. All samples were degassed at 373 K under a constant flow of N_2_ overnight.

X-ray photoelectron spectroscopy (XPS) measurements were performed at the Ambient Pressure X-ray Photoelectron Spectroscopy (APXPS) beamlines 9.3.1 and 9.3.2, at the Advanced Light Source (ALS). The Mo-doped sample was measured at Beamline 9.3.1, while the V-doped sample was measured at Beamline 9.3.2. Beamline 9.3.1 is a tender X-ray facility (photon energy range: 2.0–6.0 keV) equipped with a bending magnet and a Si (111) double-crystal monochromator.^26^ Measurements were conducted at photon energies of 3400, 4000, and 5000 eV in normal emission geometry, with a pass energy of 100 eV using an R4000 HiPP-2 Scienta analyzer. The incident beam spot size was ∼350 µm in diameter, and the sample manipulator provided four-axis control (*x*, *y*, *z*, and *θ*). Beamline 9.3.2 is a soft X-ray facility covering photon energies from 200 to 900 eV.^27^ Measurements were performed at 570, 610, 730, 810, and 890 eV, with a pass energy of 100 eV for all energies except for 570 eV, where 50 eV was used. The X-ray spot size was 1.0 mm in diameter, incident at 15° relative to the sample surface.

All XPS measurements at Beamline 9.3.2 were carried out at room temperature under ultra-high vacuum (UHV, ∼10^−9^ Torr) conditions, whereas measurements at Beamline 9.3.1 were conducted under high-vacuum conditions (HV, ∼10^−2^ Torr). The binding energy (BE) scale was calibrated against the Au 4f_7/2_ core-level peak (BE = 84.0 eV). Samples were prepared by the drop-cast method. Briefly, 30 mg of the as-prepared powder was dispersed in 6 mL of a DI water/ethanol mixture (1 : 2 volume ratio; 2 mL DI water and 4 mL ethanol) and ultrasonicated for 30 min to achieve uniform dispersion. The resulting suspension was then drop-cast onto an Au-coated Si wafer and dried at room temperature.

### 2.4 Chemical reduction and hydrogen transfer reactions

Hydrogen tungsten bronze materials (H_*z*_Mo^6+^:WO_3_ and H_*z*_V^5+^:WO_3_) were prepared *via* a solution-based reduction process previously developed by our group.^13^ Briefly, 0.2375 g of the oxide materials were added into a 20 mL scintillation vial along with 312 µL of H_2_PtCl_6_ (5% mass loading with respect to TMO), 5 mL of DI water, a magnetic stir bar, and 300 µL of H_3_PO_2_. The reaction mixtures were capped and heated between 95 °C and 100 °C in an oil bath with constant magnetic stirring at 500 rpm for 2 hours, after which they were allowed to cool to room temperature naturally with continued stirring. During the heating process, the yellow powders gradually turned blue indicating reduction of the transition metal ions. After the reaction, the samples were transferred to 50 mL centrifuge tubes and centrifuged at 4500 rpm for 5 minutes. After this, the supernatant solution was discarded and the solid was resuspended in 30 mL of H_2_O before being centrifuged again. This process was repeated two more times with the final wash using acetone. The samples were then dried at room temperature under vacuum overnight. After drying, samples were stored in a N_2_-filled glovebox (MBraun, <0.5 ppm O_2_ and <0.5 ppm H_2_O) to prevent oxidation.

For hydrogenation reactions involving TEMPO, approximately 50 mg of the hydrogen bronze powders were added into a 20 mL scintillation vial along with a magnetic stir bar and 7 mL of a 0.05 M solution of TEMPO in toluene. The contents were capped and allowed to magnetically stir overnight at room temperature. After stirring, the solutions were transferred to 10 mL centrifuge tubes and centrifuged at 4500 rpm for 5 minutes to separate the solid from the TEMPO solution.

UV-Vis-NIR spectroscopy data of the supernatant TEMPO solutions were collected on an Ocean-Insights HDX-XR spectrometer equipped with an HL-2000-HP-FHSA light source, a SQUARE-ONE cuvette holder, and QP600-1-VIS-NIR fiber optic cables. For measurements, 2.5 mL of the TEMPO supernatant solutions were added to a quartz cuvette. Scans were collected with 100 ms integration times and averaged over 15 scans with a boxcar width of 5. A calibration curve was constructed using solutions with known concentrations of TEMPO (0.10, 0.07, 0.05, 0.03, and 0.01 M) in toluene. The change in absorbance intensity at 470 nm was fit to a linear regression trend following Beer's law, absorbance = *εbc*, where *ε* is the molar absorption coefficient of TEMPO (slope of the trend line), *b* is the pathlength of the cuvette used (1 cm) and *c* is the concentration of the solution. The absorbance spectra collected for the TEMPO reactions with the bronze phases could then be used to determine the extent of conversion of optically active TEMPO to inactive TEMPOH.

### 2.5 Electrode preparation

For electrochemical measurements, slurry-cast composite electrodes on carbon paper (Fuel Cell Earth) substrates were prepared from the as-synthesized powder samples. The carbon paper substrates were cleaned by O_2_ plasma treatment for five minutes before the composite slurries were deposited on the substrate. First, 10 mg of PVDF was dissolved in 500 µL of NMP. Next, 80 mg of the powder sample was ground together with 10 mg of acetylene black for five minutes with acetone used as a grinding medium. The solvent was evaporated, and the homogeneous powder mixture was added to the PVDF/NMP solution. The resulting mixture was then stirred for six minutes using a Thinky Mixer (Thinky U.S.A.) at 2000 rpm to create a viscous ink. 7 µL of the slurry ink were then pipetted onto half of a 2 cm^2^ carbon paper electrode giving an approximate mass loading of 1 mg of active material per 1 cm^2^ of electrode area. The electrodes were then heated overnight at 120 °C under vacuum to remove the NMP solvent. For electrochemical analysis in non-aqueous electrolytes, the slurry-cast composite electrodes were prepared in the same manner but were deposited onto stainless steel mesh substrates (SS304 woven, Fisher Scientific).

### 2.6 Electrochemical characterization

Cyclic voltammetry (CV) experiments were performed on a BioLogic MPG2 potentiostat. The three electrode cells used for electrochemical analysis consisted of the as-prepared carbon paper working electrodes suspended vertically in a three-neck flask with a Pt wire counter electrode (Sigma-Aldrich), a Ag/AgCl reference electrode (4 M KCl, Pine Research Instrumentation), and 20 mL of a 0.5 M H_2_SO_4_ (pH = 0.30) solution in DI water serving as the electrolyte. The pH of the electrolyte solution was adjusted by the dropwise addition of a 1 M NaOH solution in DI H_2_O. A pH probe (Mettler Toledo, FiveEasy) was used to measure the pH of the electrolyte solution. The electrodes were allowed to soak in the electrolyte within the cell for 30 minutes prior to cycling to ensure proper wetting of the carbon paper substrate. CV experiments were conducted from −0.4 V to +0.5 V *vs.* the Ag/AgCl reference (unless otherwise noted) with scan rates of 100, 50, 10, 5, and 1 mV s^−1^. The specific capacity and subsequent proton content were determined by integrating the current response (corrected for active material mass loading) with respect to time. The potential scale was converted to the reversible hydrogen electrode (RHE) scale using the equation*E*(*vs.* RHE) = *E*(Ag/AgCl) + 0.059 × (pH) + 0.197

Non-aqueous CV experiments were performed in an argon filled glove box (MBraun, O_2_ < 0.5 ppm, H_2_O < 0.5 ppm) using a BioLogic VMP3 potentiostat. Measurements were conducted in three electrode cells consisting of the as-prepared stainless steel working electrodes suspended vertically in a three-neck flask with Li metal serving as both the counter and reference electrodes and 20 mL of a 1 M LiPF_6_ in EC/DMC serving as the electrolyte. CV experiments were conducted from 4.0 V to 1.5 V *vs.* the Li-metal reference with a 1 mV s^−1^ scan rate. The specific capacity and subsequent Li-content were determined by integrating the current response (corrected for active materials mass loading) with respect to time. The potential range was converted to the RHE scale using the following equation:*E*(*vs.* RHE) = *E*(*vs.* Li/Li^+^) − 3.040

### 2.7 *Operando* structural characterization

*Operando* electrochemical X-ray powder diffraction (EC-XRD) measurements were conducted at the Stanford Synchrotron Radiation Lightsource (SSRL) using BL 2-1 in Bragg–Brentano geometry with an incident energy of 17 keV and a Pilatus 100 K area detector was used to collect the area diffraction patterns. The 2D diffraction patterns were compiled and integrated using a Python script developed at SSRL beamline 2-1. Diffraction patterns were collected from 10° to 20° 2*θ* with a fixed incident angle (*θ*) of 4°. For direct comparison with laboratory diffraction measurements all angular ranges were converted to the scattering vector, *Q*, in reciprocal space (Å^−1^) using the equation *Q* = (4π/*λ*) × sin (2*θ*/2). All *operando* measurements were performed in an *in situ* cell following previously described protocols.^4,28^ The cell was composed of a 5.0 mm thick PEEK electrode holder with a 1/32″ thick fluorosilicone rubber gasket (McMaster-Carr) for preventing electrolyte leaks and a polyimide (Kapton) film to provide a low background window suitable for X-ray transmission. The *in situ* cell was assembled and then placed in a helium (He) chamber. The full assembly was mounted onto the diffractometer. For *operando* EC-XRD measurements, slurry-cast composite electrodes were deposited onto titanium mesh (gauze, 100 mesh, ThermoFisher Scientific) substrates with dimensions of 40 mm *×* 5 mm. For these electrodes, the mass loading was increased to approximately 5 mg cm^−2^ to improve the diffraction statistics. For measurements, cyclic voltammetry was performed in the *in situ* cell at a scan rate of 0.5 mV s^−1^ for two cycles from −0.38 V to +0.4 V (*vs.* Ag/AgCl) using a BioLogic SP150 potentiostat in a three-electrode configuration with carbon paper serving as the counter electrode, a leakless Ag/AgCl reference electrode (Pine), and a 0.5 M H_2_SO_4_ solution in DI H_2_O serving as the electrolyte.

### 2.8 Density functional theory (DFT) calculations

DFT calculations were performed starting with the pristine, monoclinic WO_3_ unit cell (64 atoms) with a 2 × 2 × 2 supercell (256 atoms), which served as the basis for both the undoped and doped systems. Doping was achieved by substituting two tungsten (W) atoms with two identical dopant atoms (*e.g.* Mo, V, or Nb), resulting in doped WO_3_ supercells (3% dopant substitution). For each doped configuration, two spatial arrangements of the dopants were considered: a “near” configuration, where the dopants were separated by a single oxygen atom, and a “far” configuration, where the dopants were further spatially separated. Hydrogen intercalation was then modeled by inserting a single hydrogen atom into the supercell. In the “near” doped structures, the hydrogen atom was bound to the bridging oxygen atom between one dopant and one tungsten atom (this is the site with the lowest energy), while in the “far” doped structures, the hydrogen atom was bound to the oxygen positioned between two tungsten atoms neighboring one dopant (this is also the site with the lowest energy). To further explore defect effects, an oxygen vacancy was introduced into the non-hydrogenated structures by removing the aforementioned oxygen atoms. Subsequently, an atomic hydrogen was added to these structures positioned as described in S4. All structures were created using the Atomic Simulation Environment (ASE) library in Python and the Avogadro software.^29,30^ All generated structures were geometrically optimized while keeping the lattice parameters fixed (at the lattice of the optimized, pristine WO_3_).

The geometry optimizations of the structures were carried out using CP2K,^31^ which uses a mixed Gaussian and plane-wave approach: the wavefunctions are expanded in a Gaussian basis set, while the electronic density is represented on a plane-wave grid.^32^ The Perdew–Burke–Ernzerhof (PBE)^33^ exchange–correlation functional was used to describe electron exchange and correlation effects.^34^ Long-range dispersion interactions were accounted for with Grimme's D3 correction.^35,36^ Triple-ζ valence polarized (TZVP) basis functions [IB5] were applied to oxygen and hydrogen atoms, while double-ζ valence polarized (DZVP) basis functions [IB6] were used for tungsten, niobium, vanadium, and molybdenum.^37^ Core electrons were treated with Goedecker–Teter–Hütter (GTH) pseudopotentials.^38,39^ Geometry relaxations were performed using the Broyden–Fletcher–Goldfarb–Shanno (BFGS) algorithm,^40^ with a maximum force criterion of 0.01 eV Å^−1^, a self-consistent field (SCF) convergence threshold of 10^−7^ Hartree and a kinetic energy cutoff of 550 Ry.

Following structural optimization, Bader charge analysis was performed to calculate the net charges of the atoms of interest.^41^ Additionally, hydrogen binding energies (HBEs) were computed using the following expression:HBE = *E*_S,H_ − *E*_S_ − 1/2*E*_H_2__,where *E*_S,H_ is the total energy of the hydrogen-intercalated structure, *E*_S_ is the total energy of the corresponding non-intercalated structure, and *E*_H_2__ is the energy of an isolated H_2_ molecule. Oxygen vacancy formation energies (OVFEs) were also calculated using the following equation, which accounts for the formation of water as a reference:OVFE = *E*_S,VAC_ − *E*_S_ + *E*_H_2_O_ − *E*_H_2__,where *E*_S,VAC_ is the total energy of the structure containing an oxygen vacancy and *E*_H_2_O_ is the energy of an isolated H_2_O molecule.

## 3 Results and discussion

### 3.1 Physical characterization of heteroatom doped tungsten oxides

**Fig. 2:**
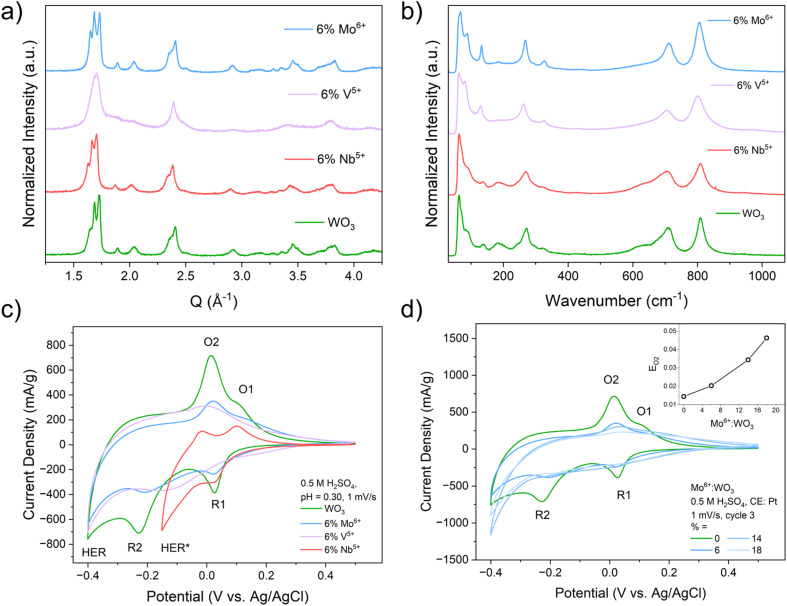
Structural characterization – PXRD (a) and Raman spectroscopy (b) – and electrochemical characterization (c) for each heteroatom dopant at a 6% concentration compared to pristine WO_3_. CV results for the Mo-series at all dopant concentrations (d); the inset figure shows the shift of the primary oxidation feature (O2) with the Mo^6+^ dopant concentration.

XRF was used to assess the incorporation of the heteroatom dopants into WO_3_ from the modified acid precipitation syntheses (Table S1). The three series of heteroatom doped WO_3_ compositions (Mo^6+^, V^5+^, and Nb^5+^:WO_3_) were all found to be isostructural to monoclinic (*γ*)-WO_3_ based on the XRD and Raman results (Fig. S1). There were more noticeable structural changes in the V^5+^:WO_3_ series due to the smaller ionic radius of V^5+^ compared to W^6+^, Mo^6+^, and Nb^5+^. Representative XRD patterns and Raman spectra for compositions with 6% dopant substitution for all three series are shown in Fig. 2a and b. SEM (Fig. S2) and BET (Fig. S3) measurements indicate similar plate-like morphologies for all compositions with the heteroatom doped samples having smaller overall particle sizes and larger surface areas compared to the pristine WO_3_. Depth-dependent XPS measurements (Fig. S4 and S5) were used to probe the distribution of the redox-active heteroatoms (Mo^6+^ and V^5+^) within the samples. The higher the XPS photon energy, the further the probing depth within the sample. These measurements show that Mo^6+^ tends to aggregate near the particle surface while V^5+^ tends to aggregate more in the sub-surface and bulk of the particle. The XPS measurements of the V^5+^ sample also show the appearance of oxygen vacancies, consistent with the decrease in the average transition metal oxidation state (Fig. S6). A more in-depth discussion on the physical characterization of these materials is provided in Discussion S1.

### 3.2 Electrochemical characterization of heteroatom doped tungsten oxides

We studied the effects of heteroatom doping on PICET in WO_3_ with cyclic voltammetry (CV) in an acidic aqueous electrolyte (0.5 M H_2_SO_4_, pH = 0.30; Fig. 2c and d). We previously reported the electrochemical PICET reactions of WO_3_.^4^ Briefly, the two sets of redox-couples observed in the CV are associated with PICET-induced structural transitions from monoclinic WO_3_ to a tetragonal (O1/R1) and cubic (O2/R2) hydrogen bronze phase, H_*z*_WO_3_, respectively. These PICET reactions occur prior to the onset of the hydrogen evolution reaction (HER), a competing PCET reaction. For the heteroatom dopants, Mo^6+^ and V^5+^ can both undergo PICET reactions in their binary oxide phases (MoO_3_ and V_2_O_5_) while Nb^5+^ (Nb_2_O_5_) does not undergo PICET prior to the onset of hydrogen evolution.^5^ For the two redox-active dopants, three main changes are observed as a function of dopant amount: (1) the potentials of the redox couples shift, (2) the peak currents decrease, and (3) the redox peaks become increasingly broader (Fig. 2c, d and S7). The Nb-doped series also shows decreased peak currents but there are only marginal, albeit negative, shifts in peak potential and markedly less peak broadening (Fig. 2c and S7). Unique to the Nb-doped series was an increase in HER activity at more positive potentials compared to WO_3_ (Fig. S7b), which made it impossible to resolve the second redox-couple (O2/R2). Because of this, the cathodic turnover potential was limited to −0.15 V (*vs.* Ag/AgCl) for slow scan rate sweeps (1 mV s^−1^ and 5 mV s^−1^). The increased HER activity of a related system, Nb-doped W_18_O_49_, was achieved previously.^42^

For the Mo-doped series, we observed a positive shift in peak potentials (Fig. 2d) suggesting that Mo-doping leads to a change in PICET thermodynamics (*i.e.*, the BDFE for hydrogen). A positive shift indicates that more energy is required to homolytically cleave the electrochemically formed O–H bond.^16^ For the most doped sample (18% Mo^6+^:WO_3_), we observed a +32 mV (+0.74 kcal mol^−1^) shift in redox peak potential compared to WO_3_. The V-doped series showed a gradual shift towards more negative potentials with 1% V^5+^:WO_3_ having a small positive shift in the O2/R2 redox-couple and no shift for the O1/R1 couple compared to WO_3_ (Fig. S7a). For the most doped sample (6% V^5+^:WO_3_), we observed a −20 mV (−0.46 kcal mol^−1^) shift in redox peak potential compared to WO_3_. A decrease in peak potential would indicate a lower BDFE or less energy required to cleave the electrochemically formed O–H bond. The potential shift observed for the Nb-doped series was significantly smaller than that for the two redox-active series, showing only a −4 mV (−0.09 kcal mol^−1^) shift at 16% Nb-doping (Fig. S7b). For all three dopant series, we observe a decrease in the number of electrochemically inserted protons/electrons compared to WO_3_ (Fig. S7c). More discussion on the scan rate dependent measurements and kinetic analysis can be found in Discussion S2, Fig S8 and S9.

The broadening of the peak-current response is only observed for redox-active dopants (Mo^6+^ and V^5+^) and increases with their amount. For PICET with WO_3_, inserted protons are localized at the bridging oxygens, forming a hydroxyl (M–OH) group. The electrochemical potential associated with PICET is thus influenced by the coordination environment of the oxygens. In doped WO_3_, assuming a random distribution of dopants, we would expect three distinct oxygen environments: (1) bridging between two tungsten sites (W–O–W), (2) bridging between a tungsten site and a dopant site (D, *i.e.*, W–O–D), and (3) bridging between two dopant sites (D–O–D). We expect that these different environments should lead to unique binding sites for inserted protons, although the extent of this difference may not be substantial enough to lead to isolated redox associated with the dopant, as observed in polyoxotungstate molecular clusters.^17,19^

### 3.3 PICET thermochemistry of heteroatom doped tungsten oxides

We determined the hydrogen bond dissociation free-energies (H BDFEs) and pH-dependent potential shifts associated with the current peaks of the PICET reactions from CV experiments in pH adjusted electrolytes (0.5 M H_2_SO_4_ + NaOH; Fig. S10 and S11). For a 1 H^+^ per 1 e^−^ reaction, the pH dependence of the half-wave potential should follow a Nernstian trend with a shift of 59 mV per pH unit.^5,16^ The BDFE(O–H) is determined from the intercept of the Nernstian trend using eqn (1).

**Fig. 3:**
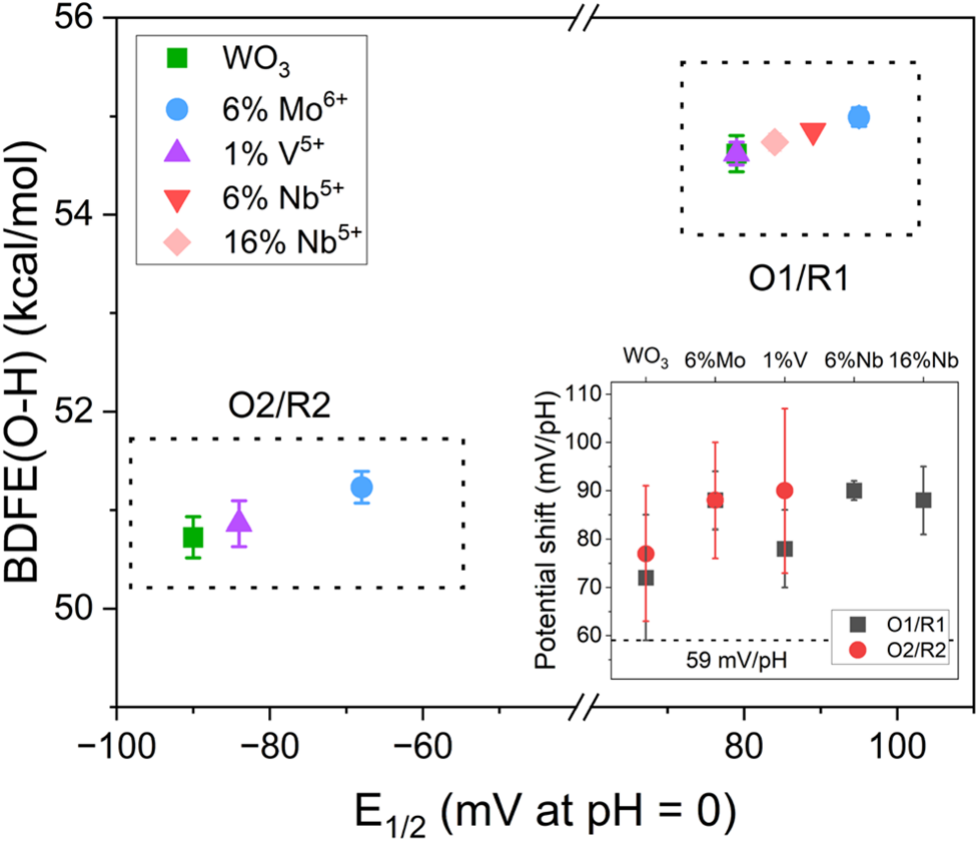
Bond dissociation free energies [BDFE(O–H)] determined for the two sets of redox peaks observed in the CV experiments (O1/R1 and O2/R2) with respect to the half-wave potential (*E*_1/2_) of the redox couples. The BDFEs for the O2/R2 couple of the Nb-doped samples could not be determined due to the increased HER activity. The inset figure shows the pH dependent potential shifts for all compounds measured with respect to the expected Nernstian shift value shown with a dashed line.

1 BDFE(O–H) = 23.06 *E*^0^_1/2_ + 52.8 kcal mol^−1^

where *E*^0^_1/2_ is the half-wave potential (*vs.* RHE) at pH = 0. It was not possible to determine the BDFE(O–H) for all heteroatom doped samples due to the current broadening or, in the case of Nb-doping, increased HER activity. The results for WO_3_ and selected heteroatom doped samples are shown in Fig. 3 for the O1/R1 and O2/R2 redox couples, where the inset figure shows the pH dependence of the half wave potentials. In all samples, the pH dependence was >59 mV per pH unit. Such “super-Nernstian” behavior in TMOs has been noted before and attributed to > 1 H^+^ per e^−^ transfer or increasing metal–oxygen bond strength with pH.^14^ The doped samples showed slightly higher pH shifts than WO_3_. Multi-electron redox is possible for both Mo^6+^ and V^5+^ transition metal cations. However, the decrease in capacities achieved with doping suggests that this isn't operational under the given conditions. For the V^5+^- and Nb^5+^-doped samples, the presence of oxygen vacancies could facilitate the adsorption of water molecules or hydronium ions at the particle surface. For both O1/R1 and O2/R2 couples, heteroatom doping shows a fine increase of the BDFE(O–H) relative to WO_3_. Of this subset of materials studied, the largest increase occurs with 6% Mo^6+^ doping, with 0.37 kcal mol^−1^ and 0.51 kcal mol^−1^ higher than those for WO_3_ for O1/R1 and O2/R2, respectively. This is similar, though milder, to the modulation of BDFE(O–H) in polyoxotungstate clusters (20 kcal mol^−1^). The presence of the single heteroatom V dopant in [VW_5_O_19_]^3−^ could “switch on” PCET reactivity [BDFE(O–H) = 64 kcal mol^−1^] relative to the homometallic tungsten cluster by increasing the basicity of the bridging oxo sites.^19^ We hypothesize that the increased BDFE(O–H) upon heteroatom doping of WO_3_ are also due to the increased basicity of bridging oxygen sites albeit to a lesser extent.

### 3.4 *Operando* electrochemical characterization of PICET-induced structural transitions

**Fig. 4:**
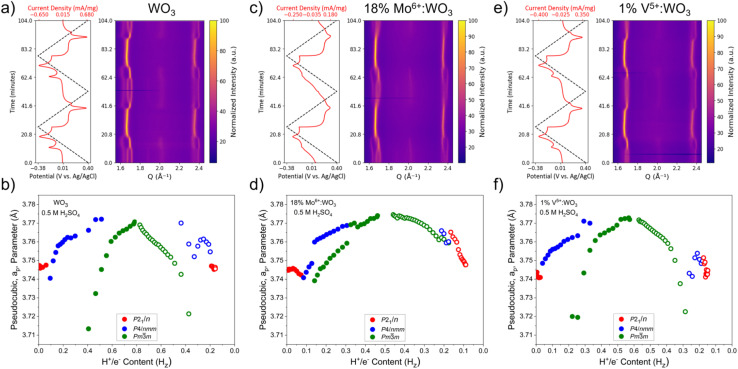
Surface contour plots showing the diffraction pattern evolution over the course of the *operando* EC-XRD measurements for WO_3_ (a), 18% Mo^6+^:WO_3_ (c), and 1% V^5+^:WO_3_ (e). Trend in pseudocubic, *a*_p_, lattice parameter with respect to the proton content for WO_3_ (b), 18% Mo^6+^:WO_3_ (d), and 1% V^5+^:WO_3_ (f). Full circles indicate the reduction sweep, and empty circles indicate the oxidation sweep for the first cycle.

The electrochemically induced structural transitions of the heteroatom doped samples were probed with *operando* electrochemical X-ray powder diffraction (EC-XRD) measurements. The PICET induced structural transitions for WO_3_ were discussed in depth previously and our results are consistent with these previous reports (Fig. 4a). The two redox features observed are associated with a transition from monoclinic (*P*2_1_/*n*) to a tetragonal bronze phase (O1/R1, *P*4/*nmm*) and a cubic bronze phase (O2/R2, *Im*3̄). The pseudocubic (*a*_p_) lattice parameter was used to evaluate the structural changes during reduction. The *a*_p_ parameter was used previously to establish lattice parameter trends in perovskite materials where the transitions are associated with changes in the octahedral tilting pattern.^4,13,43^ The equations used to calculate the *a*_p_-lattice parameter for the different structural phases are given in the SI (eqn (S1)–(S3)). The *a*_p_-lattice parameter increased upon PICET into WO_3_. Towards the apex of the redox transitions (which shows as current peaks in the CVs), WO_3_ has a biphasic mixture consisting of the two hydrogen bronze phases (Fig. 4b). This biphasic region exists only for a couple of scans at each redox event.

For 18% Mo^6+^:WO_3_ (Fig. 4c and d) which has one of the broadest current responses, *operando* EC-XRD shows more hysteresis between the structural transitions. Upon reduction, there is a prolonged biphasic region spanning 0.15 ≤ *H*_z_ ≤ 0.35, suggesting a more gradual transition than what was observed for WO_3_. Upon oxidation, the tetragonal phase is only observed very briefly within the biphasic region (0.15 ≤ *H*_z_ ≤ 0.20) and quickly devolves into the monoclinic structure. This can be visualized as the smearing of the diffraction peaks upon oxidation and from the trend in the pseudocubic axis. The cubic phase appears at significantly lower degrees of reduction (*H*_z_ = 0.15) compared to the undoped sample (*H*_z_ = 0.40).

For 1% V^5+^:WO_3_ (Fig. 4e and f) where the redox couples are still well defined, the structural transition sequence is more similar to the step-wise process observed in WO_3_ as opposed to the gradual transition observed in 18% Mo^6+^:WO_3_. However, it is again observed that the cubic phase appears at lower degrees of reduction for this sample (*H*_z_ = 0.20). We expect that the hydrogen binding affinities of the different oxygen chemical environments are enabling the structural transition to the cubic bronze phase in an effort to optimize the hydrogen bonding configuration and transport pathways within the structure. This effect would be further influenced by the amount of heteroatom dopants resulting in a greater distribution of less favorable W–O–D and D–O–D bonding environments. Similar *operando* measurements were performed for 6%V^5+^:WO_3_ and the results are discussed in Discussion S3 and Fig. S12. Previously, we pointed out that the broadened current responses observed for the more doped samples made it difficult to determine the BDFE values for the distinct PICET reactions. The *operando* EC-XRD measurements presented here further rationalize this difficulty by showing the increased coexistence of the two bronze phases in samples containing higher dopant amounts.

### 3.5 *Ex situ* characterization of heteroatom doped hydrogen bronzes

**Fig. 5:**
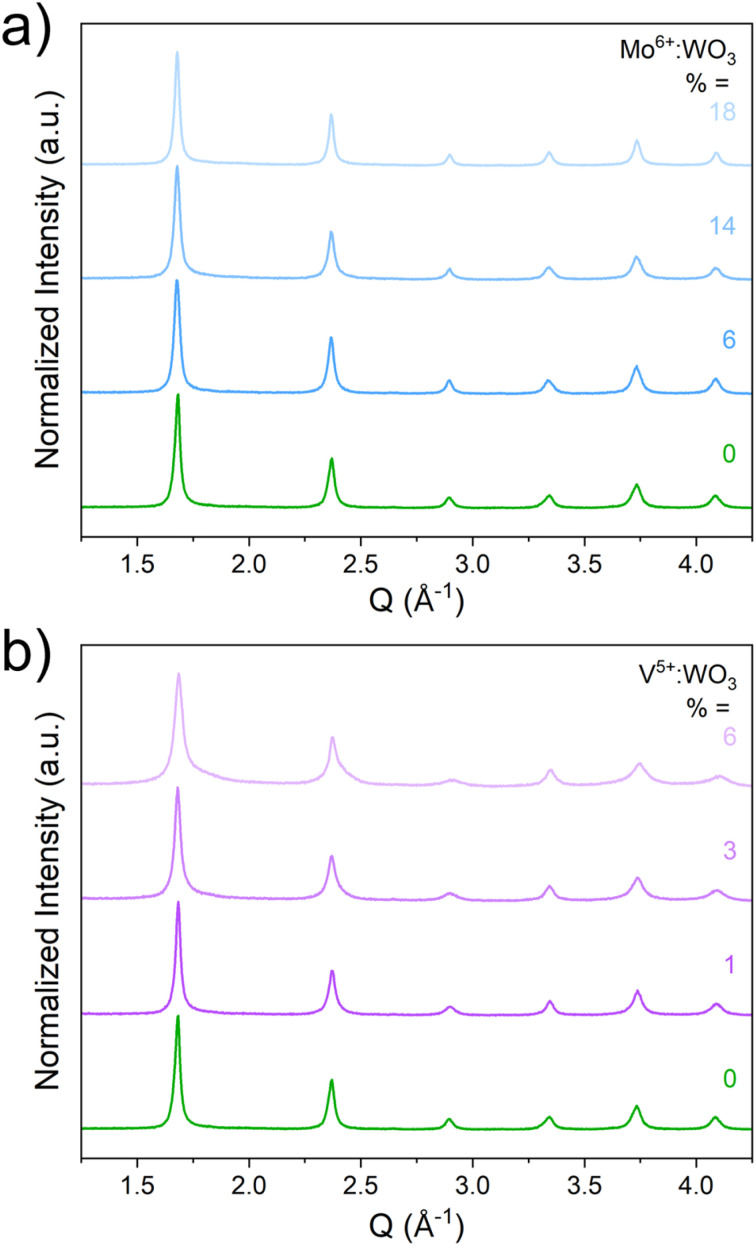
PXRD patterns for the chemically reduced tungsten oxides for the Mo-doped series (a) and the V-doped series (b).

We used chemical reduction to further assess the structures of the heteroatom doped hydrogen bronzes (H_*z*_Mo^6+^:WO_3_ and H_*z*_V^5+^:WO_3_; Fig. 5). We previously reported a solution-based method utilizing H_2_PtCl_6_ and H_3_PO_2_ to prepare bulk powders of Pt-decorated (Pt@) H_*z*_WO_3_ with the equilibrium structure and proton/electron content that would exist for an electrode polarized at 0 V *vs.* RHE.^13^ All reduced samples display a diffraction pattern consistent with the cubic bronze phase. We previously discussed that the undoped material (H_*z*_WO_3_) should have a tetragonal structure with *P*4*mm* space group symmetry. The appearance of the cubic phase for this sample is a consequence of increased preferred orientation effects hiding the tetragonal axis. This stems from the smaller particle sizes used here compared to those used in our prior report.^13^ To verify the cubic structure of the heteroatom doped samples, we conducted identical chemical reduction reactions on Mo-doped and pristine WO_3_ samples that were sintered at 500 °C for 8 hours in air to increase the average particle size (Fig. S13). For the undoped sample, the pattern displays additional peak splitting consistent with the previously reported tetragonal cell, suggesting that preferred orientation leads to the appearance of the cubic phase for this sample. For the Mo-doped samples, the diffraction patterns do not change, and the cubic structure is maintained in the larger particle size samples (Fig. S13).

We further evaluated the extent of chemical reduction and hydrogenation ability of the heteroatom doped samples using a chemical oxidant, 2,2,6,6-tetramethyl-1-piperidinyloxy (TEMPO). We previously used these reactions with TEMPO to demonstrate the ability of Pt@H_*z*_WO_3_ to perform hydrogen atom transfer (HAT) reactions and to determine the proton content of the bronze phase.^13^ The conversion of TEMPO to TEMPOH can be monitored *via* UV-Vis absorbance spectroscopy due to the change in optical response of the organic molecule upon HAT. The molar absorptivity of TEMPO in toluene was determined to be 10.22 M^−1^ cm^−1^ through linear fitting of the concentration dependent absorbance intensity curve (Fig. S14). The bronze reactions were allowed to stir overnight in a 0.05 M TEMPO in toluene solution after which the supernatants were collected fo r UV-Vis measurements. After the reaction with the bronzes, the absorbance profiles for all of the TEMPO supernatant solutions show a decrease in intensity, verifying that HAT had occurred (Fig. S15). All bronze materials were found to transfer between 0.18 and 0.28 H^+^/e^−^ per mol of TMO (Fig. S15c). The pristine sample took approximately 4 hours to return to its oxidized state, WO_3_, as determined by the visual color change from blue back to yellow. Both series of doped compounds maintained some degree of blue coloration after the overnight reaction. This may suggest that only a portion of the inserted protons are able to react with TEMPO. Given that the undoped sample was fully oxidized within 4 hours, we may expect that the most accessible protons come from protonated oxygen sites which bridge two W-centers and that protonated W–O–D and D–O–D sites are less accessible. The reduction extent of the heteroatom doped bronzes determined from the reactions with TEMPO (Fig. S15c and d) is consistent with the previous *operando* XRD results showing that the cubic bronze phase exists at lower degrees of protonation/reduction (Fig. 4).

### 3.6 Density functional theory (DFT) calculations

To further understand the role of heteroatom dopants in the PICET insertion site energetics from an atomistic level perspective, we performed DFT calculations on bulk (periodic in 3 dimensions) structures. From the experimental results (see Discussion S1) we can be confident that the proton insertion and oxygen vacancy formation (for aliovalent dopants) occurred within the bulk rather than merely at the surface of the materials, which dictates the use of periodic bulk oxide structures for the DFT calculations. First, we performed Bader charge analysis to calculate the net charges of atoms of interest in the sequence D–O–M–O/X–W–O–W, where D represents a dopant (or tungsten only for the pristine structure), M represents either a dopant or a tungsten atom (in near and far conformations respectively), and X denotes an oxygen vacancy. Regarding the stability of the far *vs.* the near configurations, the calculations showed a negligible difference in total energy, with the near configurations being less than 0.3 eV more exothermic. We further computed the hydrogen binding energies (HBEs) for all structures, both with and without dopants and oxygen vacancies. In the vacancy-containing systems, we also calculated the HBEs for hydrogen atoms located between the two metal atoms (*i.e.*, at the vacancy site as a metal hydride). Rather than calculating hydrogen interactions at different degrees of intercalation,^22^ we focused on single hydrogen atom binding energy to obtain clear trends on the binding behavior of different sites created in WO_3_ upon doping and to avoid combinatorial complexity arising from different hydrogen binding configurations in the cell. Finally, we determined the oxygen vacancy formation energies (OVFEs) for each structure. As summarized in Table S2, the net charge of the oxygen (which binds hydrogen after PICET) in the unprotonated structures is practically unaffected by heteroatom doping. The only exception to this is the near-configuration of the V-doped structure, where the first oxygen's net charge increases from −1.02*e* (in the pristine structure) to −0.81*e*. Nevertheless, we cannot observe any correlation between the net charge of oxygen and HBE. In general, no obvious correlation was found between the charge distribution on the atoms of interest and HBE.

**Fig. 6:**
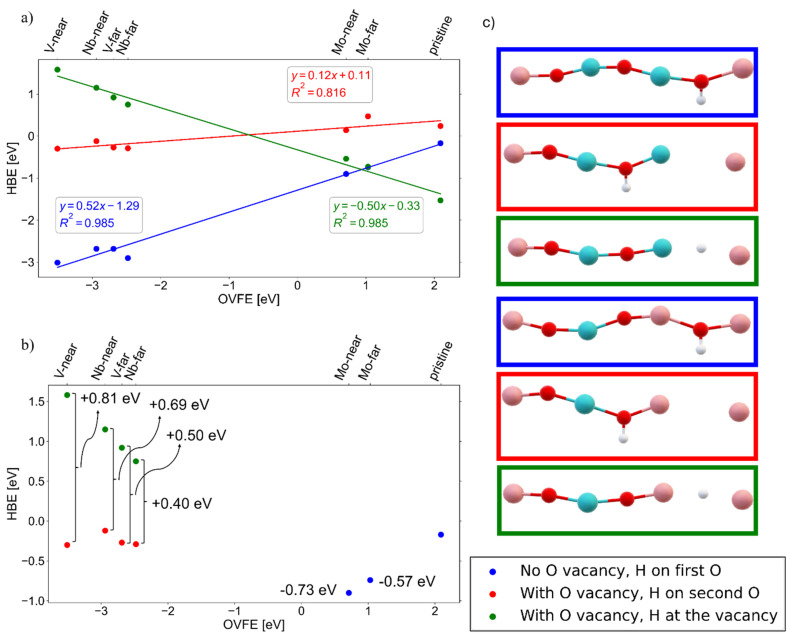
Hydrogen binding energy (HBE) as a function of oxygen-vacancy formation energy (OVFE) for different local hydrogen environments. (a) HBE *vs.* OVFE for three adsorption configurations: hydrogen bound to the middle oxygen in the D–O–M–O–W–O–W chain (no oxygen vacancy) (blue). Hydrogen bound to the left oxygen in the D–O–M–X–W–O–W chain (with an oxygen vacancy) (red). Hydrogen occupying the oxygen-vacancy site between two metals in the D–O–M–X–W–O–W chain (green). Linear fits are shown to illustrate trends. (b) Differences in HBE for experimentally observed dopant environments relative to the pristine structure. For doped systems, in which two vacancy-containing configurations are possible, the values shown represent the average HBE relative to the pristine case. (c) Schematic representations of the atomic configurations corresponding to the three cases in (a) and (b) for both near and far configurations. Cyan: dopant metal (V, Nb, or Mo), red: O, pink: W, and white: H. The bottom right legend depicts the three cases in all figures.

Regarding the HBEs, we notice that they are mostly thermoneutral (values clustered near zero) for the vacancy-containing structures (see Fig. S16). In contrast, vacancy-free structures (see Fig. S16) display very different behavior: the isovalent-doped (Mo-doped) and pristine systems exhibit mildly exothermic HBEs (*i.e.*, close to thermoneutrality), whereas the aliovalent-doped (Nb- and V-doped) systems show highly exothermic values. For hydrogen bound at the vacancy site (see Fig. S16), the trend is reversed; the isovalent-doped systems exhibit very exothermic HBEs, while the aliovalent-doped ones are endothermic. These three scenarios are illustrated in Fig. 6, where HBE is plotted as a function of OVFE.

For vacancy-free structures, HBEs show a strong linear correlation with OVFEs. A similar trend is observed for hydrogen positioned between the two metals in the vacancy-containing structures (*i.e.* as a hydride). In contrast, when hydrogen is bound to an oxygen adjacent to a vacancy, the coefficient of determination (*R*^2^) is lower, because the HBE values are practically thermoneutral (slope close to 0). Another interesting result observed is the separation of the doped structures with respect to their OVFEs. All aliovalent doped structures have significantly negative (exothermic) OVFEs whereas the isovalent and pristine ones exhibit much positive OVFEs, regardless of the position of the two dopants. The DFT results suggest that the oxidation state of the dopants affects HBE the most, since the differentiation is between aliovalent and isovalent doped structures. Net charges of all atoms in the sequence D–O–M–O/X–W–O–W do not seem to play any significant role in the HBEs.

Previously, we hypothesized that the broadening of the peak-current response in the CV measurements was attributed to the distribution of oxygen chemical environments due to the introduction of the heteroatoms. The DFT calculations validate this hypothesis by showing changes in the HBEs based on the identity of the two metals which the oxygen atom bridges. For the isovalent-doped samples, both far and near configurations show more exothermic hydrogen binding compared to the pristine structure (see Fig. 6b). Experimentally, we observe a positive shift in BDFE(O–H) which agrees with the more favorable hydrogen binding on the Mo-doped oxide. For aliovalent doped samples (V and Nb), the culmination of different available sites (*i.e.* on an oxygen site or at the vacancy site) leads to an overall decrease in HBEs (weaker binding) for the different configurations, consistent with the shifts to more negative potentials observed experimentally. Between the Mo- and V-doped calculations, the relative change in HBEs indicates that V-doping would have a more drastic impact on proton binding site distribution. This matches our experimental results showing similar degrees of peak-current broadening for 6%V^5+^:WO_3_ and 18%Mo^6+^:WO_3_. For the Nb-doped series, it was computationally found that the oxygen near the vacancy is the most favorable hydrogen insertion site. The neighboring vacancy sites stabilize hydrogen as hydrides with binding energies that are less exothermic than the corresponding V-sites. As a result, the Nb-doped structure is the only oxide that stabilizes vacancies and exhibits more balanced energetics of hydrogen interaction with both bridging oxygens and neighboring vacancies (less energetic variations). This may potentially explain the increased HER activity observed experimentally, involving vacancy-sites where protons couple with hydrides on the surface, since there is no evidence of the HER occurring in the bulk.^4^ These differences in hydrogen binding site energies may also have an impact on the solid-state proton transport, restricting diffusion to the lower energy pathways. Overall, the computational results match exceptionally well with the experimental results, highlighting the importance of dopant configuration within the crystal structures.

**Fig. 7:**
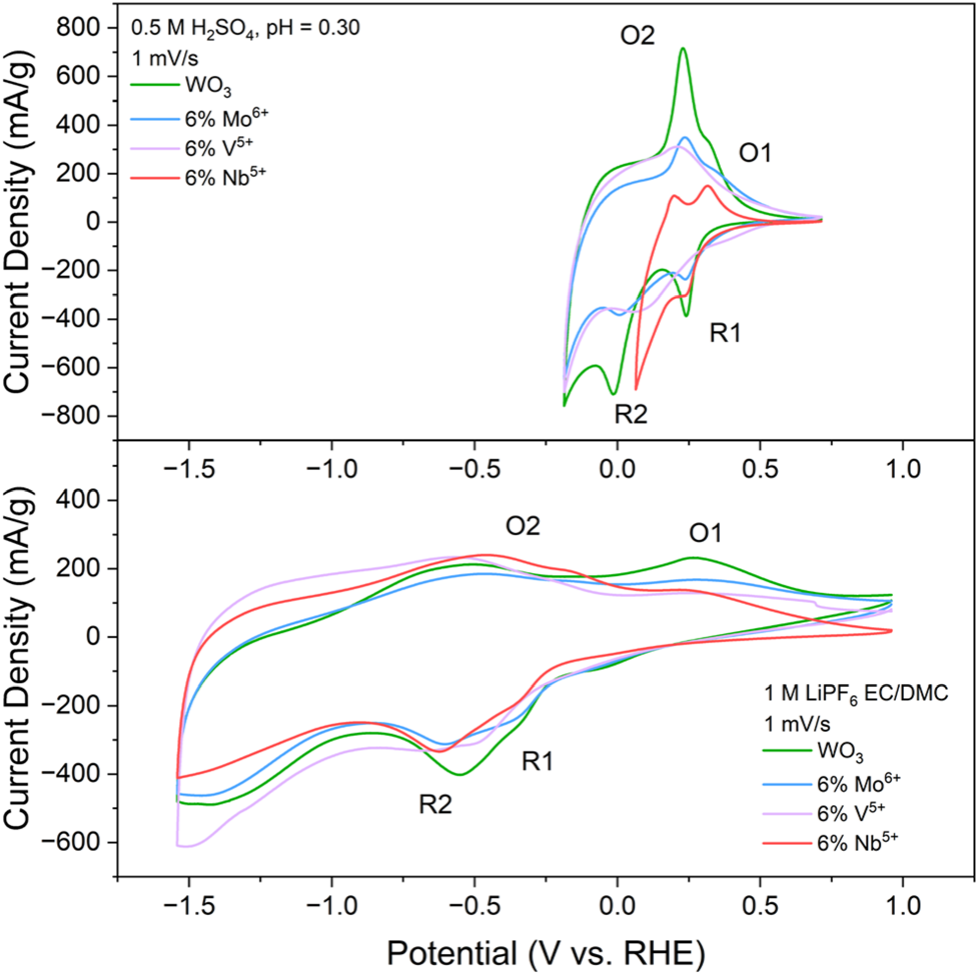
Comparison of CVs at 1 mV s^−1^ collected in 0.5 M H_2_SO_4_ (top) and 1 M LiPF_6_ in EC/DMC (bottom) for WO_3_ and the 6% heteroatom doped compositions.

While PICET is sensitive to the local oxygen environment, cation insertion-coupled electron transfer is less localized. Intercalated alkali cations such as Li^+^ reside in interstitial void spaces governed by the overall crystal structure and are largely agnostic to the specific chemical coordination environments of the oxide ligands. Therefore, cycling the heteroatom doped WO_3_ samples in a non-aqueous alkali cation-containing electrolyte should not result in the peak current broadening observed in the acidic aqueous electrolyte. To test this hypothesis, we performed CV of WO_3_ and the 6% doped samples in a Li^+^-containing electrolyte (Fig. 7). Our results support this hypothesis by showing only marginal changes in current magnitude with no significant peak broadening during electrochemical Li^+^ insertion. The 6% V-doped sample is the only composition that shows a minor shift of the redox couples to more negative potentials, which can be attributed to the previously discussed increased structural strain (*i.e.*, more different interstitial void-site volume). These experiments verify that the peak broadening observed in the aqueous electrolyte is attributed to the increased distribution of oxygen environments.

## 4 Conclusions

We report the effect of isovalent (Mo^6+^) and aliovalent (V^5+^ and Nb^5+^) doping in monoclinic (*γ*) WO_3_ on the thermodynamics of electrochemical PICET. Mo- and Nb-doped samples show no significant change in the crystal structure, while V-doping leads to broadening of the diffraction peaks consistent with increased structural strain due to the change in ionic radii. Electrochemical cycling of samples containing redox-active dopants (Mo^6+^ and V^5+^) in 0.5 M H_2_SO_4_ shows systematic shifts in the half-wave potentials for the two distinct redox couples (O1/R1 and O2/R2) and a broadening of the potential-dependent current response that increases with the dopant concentration. The incorporation of a non-redox-active dopant (Nb^5+^) considerably lowers the current response associated with PICET while increasing HER activity, with no substantial shift in redox-peak potential. The BDFE(O–H) values and pH-potential shifts for the doped and undoped samples were determined from pH-dependent CV. The sequence of PICET-induced structural transitions was probed with *operando* EC-XRD conducted in an acidic electrolyte. The broadening of the current response in the CV experiments corresponds with changes in the degree of reduction at which the hydrogen bronze phases appear and the duration of the bronze structural regimes. The presence of the heteroatom dopants changes the chemical distribution of bridging oxygen sites, finely altering the kinetics and thermodynamics of electrochemical PICET. DFT calculations support this hypothesis by showing changes in hydrogen binding energies relative to WO_3_ based on the identity of metal atoms with which the oxygen/vacancy site bridges, showing more of an impact in V-doped systems than in Mo-doped systems. DFT calculations further revealed a linear correlation between the hydrogen binding energies and oxygen vacancy formation energies for both doped and undoped structures. This correlation was observed in cases without oxygen vacancies and when an inserted proton was positioned between two metal atoms in the presence of an oxygen vacancy. These findings suggest that the oxygen vacancy formation energy may serve as a reliable descriptor for hydrogen binding energy. The combination of experimental and computational results presented here highlights the importance of location and distribution of dopants/defects in the thermodynamics of hydrogen atom transfer reactions involving redox-active metal oxides.

## Author contributions

N. P. H.: conceptualization, investigation, formal analysis, methodology, writing, review & editing; N. E. P.: investigation, formal analysis, methodology, writing, review & editing; J. R. P.: investigation, formal analysis, methodology, writing, review & editing; G. M.: funding acquisition, supervision, resources, writing, review & editing; E. J. C.: funding acquisition, supervision, resources, writing, review & editing; V. A.: funding acquisition, resources, conceptualization, supervision, writing, review & editing.

## Conflicts of interest

There are no conflicts to declare.

## Supplementary Material

SC-OLF-D5SC08564K-s001

## Data Availability

Data from this work are openly available at https://doi.org/10.5281/zenodo.17402112. Supplementary information (SI): amounts of reagents used for the synthesis of the heteroatom doped tungsten oxides (Table S1). Physical characterization discussion (Discussion S1). XRD and Raman spectra of all heteroatom doped samples (Fig. S1). SEM images of the heteroatom doped and undoped WO_3_ samples (Fig. S2). BET sorption measurements and determined surface areas of the heteroatom doped and undoped WO_3_ samples (Fig. S3). Synchrotron XPS characterization results for Mo^6+^ (Fig. S4) and V^5+^ (Fig. S5 and S6). CV results at 1 mV s^−1^ for the aliovalent doped series in 0.5 M H_2_SO_4_ (Fig. S7). Discussion on scan rate dependence (Discussion S2). Scan rate dependent CV experiments for all compositions (Fig. S8). *B*-value analysis of the scan-rate dependent CV measurements (Fig. S9). Variable pH CV measurements and analysis of undoped and Mo/V-doped compositions (Fig. S10). Variable pH CV measurements and analysis of the Nb-doped compositions (Fig. S11). Equations used to calculate the pseudocubic lattice parameters (eqn (S1)–(S3)). Discussion on 6% V^5+^:WO_3_*operando* EC-XRD measurements (Discussion S3). Surface plots and pseudocubic lattice parameter trends for 6% V^5+^:WO_3_ from *operando* EC-XRD measurements in 0.5 M H_2_SO_4_ (Fig. S12). PXRD patterns for the chemically reduced Mo-doped samples with larger particle sizes (Fig. S13). Concentration dependent absorption measurements and calibration curve for TEMPO in toluene (Fig. S14). TEMPO chemical oxidation and UV-Vis results (Fig. S15). DFT result discussion (Discussion S4). DFT calculations for net charges, HBEs, and OVFEs (Table S2). Visualization of the atoms of interest for the 3 cases of proton binding (Fig. S16). See DOI: https://doi.org/10.1039/d5sc08564k.
