# Supplementary material for: Effects of heteroatom doping on hydrogen uptake in tungsten oxide
Source: Chem Sci. 2026 Jan 15;17(9):4548–61. doi: 10.1039/d5sc08564k (PMC12805440; doi:10.1039/d5sc08564k)
Supplement: SC-017-D5SC08564K-s001 [file SC-017-D5SC08564K-s001.pdf]

Supplementary Information (SI) for Chemical Science.
This journal is © The Royal Society of Chemistry 2025

Supplemental Information for:

Role of Heteroatom Doping on Hydrogen Uptake in Tungsten Oxide

Noah P. Holzapfel[1], Nikos Papamatthaiakis[2], Jay R. Paudel[3], Giannis Mpourmpakis[2,4], Ethan J. Crumlin[3,5], and Veronica Augustyn[1]*

[1]Dept. of Materials Science and Engineering, North Carolina State University, Raleigh, NC 27695, USA

[2]School of Chemical Engineering, National Technical University of Athens (NTUA), Athens GR-15780, Greece

[3]Chemical Sciences Division, Lawrence Berkeley National Laboratory, Berkeley, CA 94720, USA

[4]Department of Chemical and Petroleum Engineering, University of Pittsburgh, Pittsburgh, PA 15261, USA

[5]Advanced Light Source, Lawrence Berkeley National Laboratory, Berkeley, CA 94720, USA

* corresponding author e-mail: vaugust@ncsu.edu

Table S1: Synthesis conditions for the three series of heteroatom doped WO_3 samples. Mo- and Nb-doped samples were prepared at room temperature. V-doped samples were heated to 80°C to ensure complete dissolution of V-precursor before cooling to room temperature prior to acid precipitation.

Mass $Na_2WO_4{\cdot}H_2O$ (g)	Mass $Na_2MoO_4{\cdot}H_2O$ (g)	Expected Mo mol %	Volume H_2O (mL)	Volume 6 M HNO_3 (mL)	% Dopant In Product (XRF)
1.6493	0.0605	5	2.5	10	6
1.6493	0.1452	11	2.5	10	14
1.6493	0.2057	15	2.5	10	18
Mass $Na_2WO_4{\cdot}H_2O$ (g)	Mass $NaVO_3$ (g)	Expected V mol %	Volume H_2O (mL)	Volume 6 M HNO_3 (mL)	% Dopant In Product (XRF)
1.6493	0.0122	2	5	10	1
1.6493	0.0244	4	5	10	3
1.6493	0.0610	9	5	10	6
Mass $Na_2WO_4{\cdot}H_2O$ (g)	Mass $NbCl_5$ (g)	Expected Nb mol %	Volume H_2O/EtOH (mL)	Volume 6 M HCl (mL)	% Dopant In Product (XRF)
1.6493	0.0675	5	2/1	10	6
1.6493	0.2026	15	2/1	10	16

Discussion S1:

Physical Characterization of Heteroatom Doped Tungsten Oxides

We used XRF to determine the dopant amounts incorporated into WO_3 from the modified acid precipitation syntheses. The Mo- and Nb-doped samples had concentrations close to the desired stoichiometry while the V-doped samples had notably lower amounts than expected (**Table S1**). We attribute this to two factors: (1) the lower aqueous solubility of $NaVO_3$ relative to Na_2WO_4 and Na_2MoO_4 (0.193 vs. 0.742 and 0.76 g/mL, respectively) and (2) the higher solubility of $NaVO_3$ in acidic aqueous solutions relative to $WO_3 \cdot 2H_2O$, which readily precipitates in acid.

The phase purity and crystal structures of the heteroatom doped WO_3 samples were assessed via powder X-ray diffraction (PXRD). For the Mo^{6+}:WO_3 series (% = 6, 14, and 18) and the Nb^{5+}:WO_3 series (% = 6 and 16), all compositions were isostructural with γ-WO_3 which has monoclinic $P2_1/n$ space group symmetry (**Fig. S1**). We did not observe any significant changes in peak position upon Mo- or Nb-doping, which may be attributed to the similar ionic radii of W^{6+} (0.60 Å), Mo^{6+} (0.59 Å), and Nb^{5+} (0.64 Å) in a 6-coordinate environment.[1] The WO_3–MoO_3 phase diagram shows that the γ-WO_3 structure exists up to 23% Mo substitution and is consistent with our observations here.[2] A previous report on the Nb_2O_5–WO_3 solid-solution describes some uncertainty in the phases which exist below 20% Nb-substitution.[3] However, this previous study utilized high temperature solid-state reactions to produce the thermodynamically favored phases as opposed to the solution-based method we used, which may produce a kinetically favored solid-solution phase. Both the Mo- and Nb-doped samples were slightly light sensitive and would photoreduce upon prolonged exposure to ambient light or X-rays, as indicated by a color change of the yellow powders to blue. This photoreduction may involve the adsorption and volatilization of in situ generated water (Nb/Mo/W-OH_2) at the particle surface, leading to a partially oxygen deficient surface (Mo^{6+}/Nb^{5+}:WO_{3-y}).[4,5] As a result, the Mo- and Nb-doped samples were heated at 120°C in air prior to all physical and electrochemical characterization. Sample oxidation was visually assessed by the color change back to yellow.

For the vanadium-doped samples (V^{5+}:WO_3, V:W % = 1, 3, and 6) the diffraction peaks increasingly broaden with increasing doping amount and partially merge together. However, the reflections are still consistent with that of γ-WO_3 with the lattice parameters becoming more similar in length (**Fig. S1**). We expect the smaller ionic radius of V^{5+} (0.46 Å in a 5-coordinate geometry or 0.54 Å in a 6-coordinate environment) to induce some strain on the WO_3 structure, leading to the observed XRD peak broadening. It also follows that such strain broadening would increase with the amount of V^{5+}-doping. Previous reports on the V_2O_5–WO_3 solid solution indicates a tetragonal phase near the solubility limit of 10 % V-substitution.[6,7] The lack of a tetragonal phase in our PXRD results suggests that we are below this limit, consistent with the dopant concentrations determined from XRF (6% in the most doped sample). Similar to the Nb-doped series, solution-based and ceramic synthetic strategies may lead to different structural polymorphs of the same composition. The V-doped samples were not sensitive to light or X-rays, as indicated by the lack of color change upon exposure. We used Raman spectroscopy to further verify the structural similarities of the doped samples with WO_3 (**Fig. S1**). For all heteroatom doped samples, the Raman spectra share similar features with γ-WO_3. This confirms that the local structure of the WO_3 host was maintained upon both iso- and aliovalent doping at all levels explored here.

We performed SEM and BET analysis to determine the effects of heteroatom doping on the particle microstructure. Undoped WO_3 had a nanoplatelet morphology consistent with previous reports utilizing the acid precipitation and thermal dehydration synthesis method (**Fig. S2**).[8,9] SEM images of the doped samples show similar nanoplatelet morphologies as WO_3 but with notably smaller sizes (**Fig. S2**). Consistent with the observations from SEM images, the BET-determined surface areas of the heteroatom doped samples were approximately twice to three-times as large, ranging from 29.14 m^2/g for 18% Mo^{6+}:WO_3 to 46.40 m^2/g for 16% Nb^{5+}:WO_3 while that of pristine WO_3 was 16.64 m^2/g; (**Fig. S3**).

We characterized the depth-dependence of the dopant concentration of selected compositions by performing synchrotron XPS measurements while varying the incident photon energy (PE) The results for 18% Mo^{6+}:WO_3 and 6% V^{5+}:WO_3, the highest doped samples in their respective series, are shown in **Figure S4** and **S5**. For 18% Mo^{6+}:WO_3, we analyzed the Mo $2p_{1/2}$ and W $3p_{1/2}$ peaks due to their spectral proximity which minimized the synchrotron data collection time. We calculated the amount of Mo and W present by integrating the areas under each peak. Varying the PEs to 3.4 keV, 4.0 keV, or 5.0 keV leads to penetration depths of 4.32 nm, 6.66 nm, and 10.26 nm, respectively. Our results show that as the probing depth increased, the fraction of Mo decreased from 27% to 18%. This suggests that the Mo-dopant tended to aggregate on the particle surface and did not have a homogeneous distribution throughout the particle. Therefore, while the XRF results showed that the overall sample composition for the Mo-doped samples was close to that expected based on the synthesis stoichiometry, the XPS results show that the Mo-dopants are not homogeneously distributed within the particles.

Similar XPS measurements could not be performed on the V-doped samples at the tender X-ray beamline due to the low photoionization cross section of the V *2p* transitions within the photon energy (PE) range accessible to the instrumentation (2.0–6.0 keV). To overcome this limitation and enable reliable detection of the V *2p* signal in V:WO_3, measurements were conducted at the soft X-ray beamline (BL 9.3.2), where the photoionization cross section for V *2p* is significantly higher. Prior to the measurements, the sample was annealed at 400 °C for 1 hr in 100 mTorr O_2 to remove the surface contamination layer. This step was essential because the reduced probing depth at soft X-ray energies increases surface sensitivity, and surface contaminants— particularly oxide-related features in the higher binding energy region of the O 1s spectrum (~531–532 eV)—can overlap with the O 1s signal from oxygen atoms near vacancy sites, could obscure or distort quantitative analysis. XPS spectra were then acquired at photon energies of 570, 610, 730, 810, and 890 eV to examine the depth-dependent distribution of V, as shown in **Figure S5**. The V concentration profile was evaluated by analyzing the area ratio of the

V $2p_{3/2}$ peak to the total O *1s* signal. The results indicate that the V concentration increases with increasing photon energy, as reflected by the progressive rise in the V $2p_{3/2}$/O *1s* area ratio, at higher photon energies. Nevertheless, even at the highest photon energy used (890 eV), which corresponds to an approximate probing depth of ~2.8 nm, the V/O ratio remained well below the theoretical value of 0.02—although it was nearly twice the value obtained at the most surface-sensitive condition (570 eV). This observation suggests that V preferentially resides in the subsurface layers, whereas Mo tends to accumulate at the surface relative to the bulk, indicating contrasting distribution behaviors for aliovalent and isovalent dopants in WO_3. In addition to the depth-dependent distribution of dopants across the surface and subsurface regions, a noticeable intensity was observed in the higher binding energy component of the O *1s* spectrum for the V-doped sample (**Figure S5a**). This peak can be attributed to oxygen atoms located near vacant sites.[5] While adventitious carbon oxides may overlap with this feature, their contribution is expected to be minimal. This is supported by the negligible C *1s* intensity relative to O *1s*, owing to the in situ annealing treatment employed to remove surface carbon. Nevertheless, a small amount of residual carbon was still detected in the survey spectrum (**Figure S6a**), which may partially contribute to the higher binding energy O 1s signal. Aside from this minor contribution, the peak is primarily assigned to oxygen atoms near vacancies.

Furthermore, the high-resolution W 4f spectrum (**Figure S6b**) indicates that tungsten is not fully in the 6+ oxidation state; approximately 5% of W exists in the 5+ state. This reduction of W from its fully oxidized state is likely associated with the presence of oxygen vacancies, consistent with the non-zero intensity of the higher binding energy O *1s* peak. Together, these observations support the interpretation that aliovalent V doping induces oxygen vacancies in WO_3. Since V is enriched in the subsurface region relative to the surface, measurements of the W electronic states were performed using a photon energy of 890 eV to enhance the probing depth and confirm the role of V in oxygen vacancy formation.

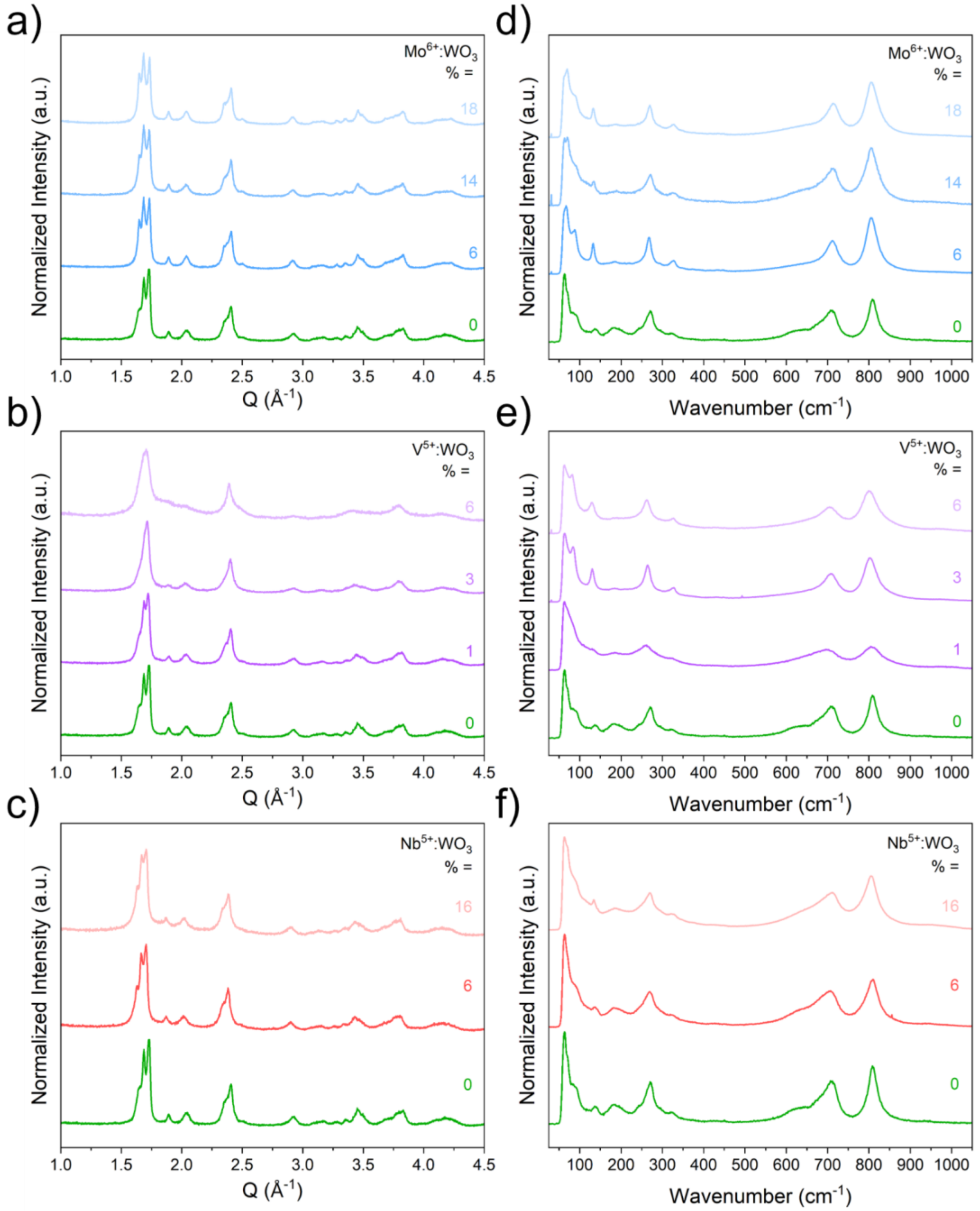

Figure S1: Stacked PXRD data (a, b, c) and Raman spectra (d, e, f) for all of the heteroatom doped compositions: Mo^{6+} (a, d), V^{5+} (b, e), and Nb^{5+} (c, f) compared to WO_3, respectively.

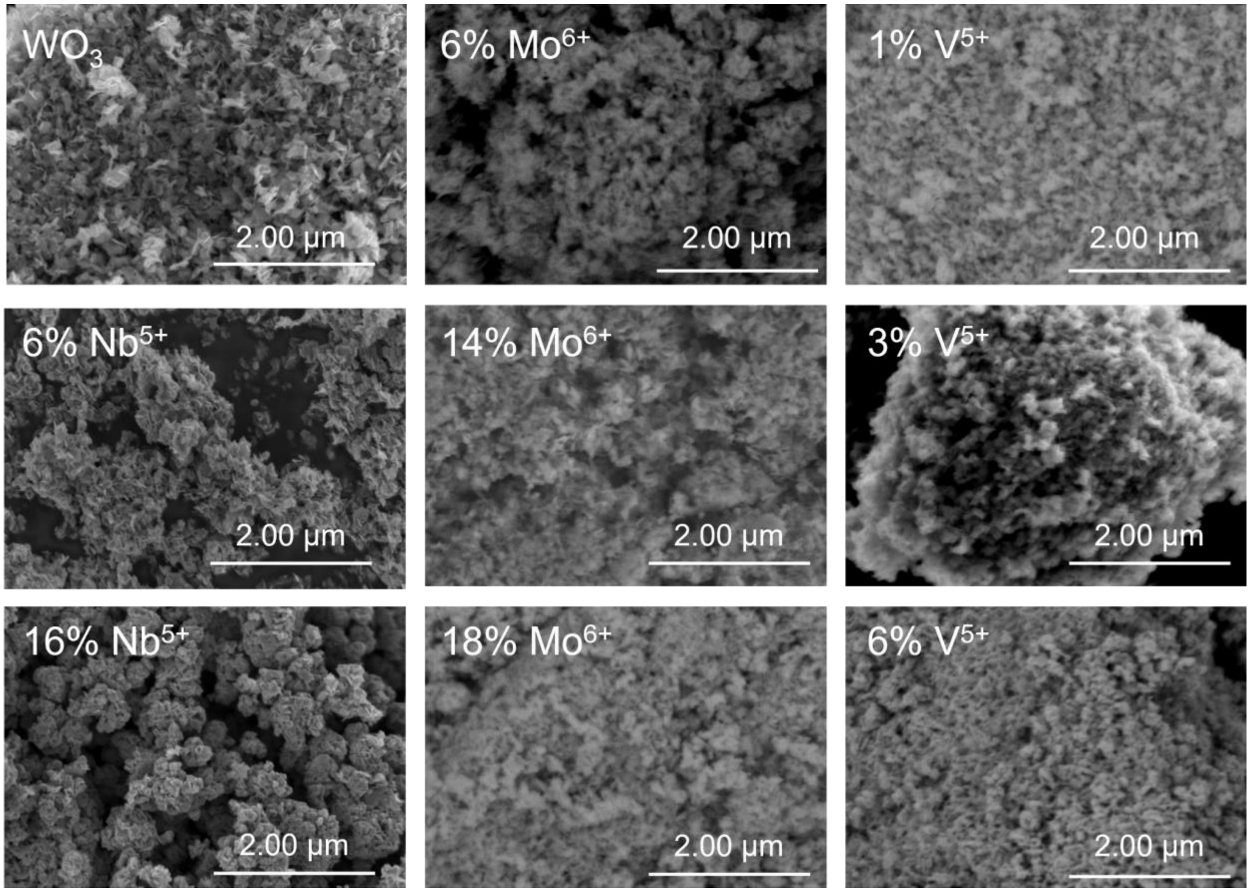

Figure S2: SEM images taken for all compositions at the same level of magnification and scale. The composition is provided with the respective micrograph.

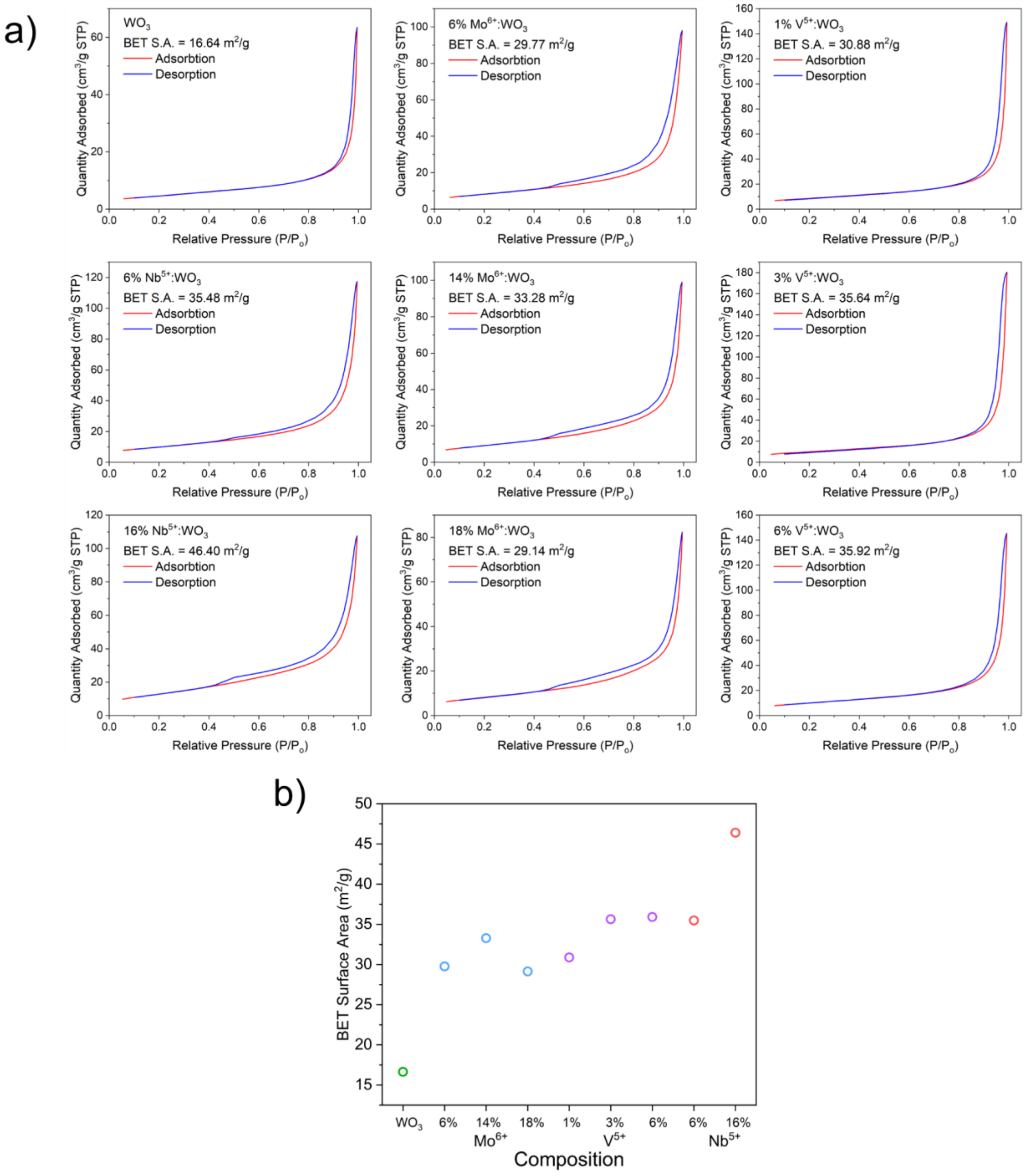

Figure S3: Individual BET sorption measurements for pristine WO_3, and the heteroatom doped samples (a). Determined surface areas (S.A.) given in figure with respective composition. Comparison of measured BET surface areas for all compositions studied (b).

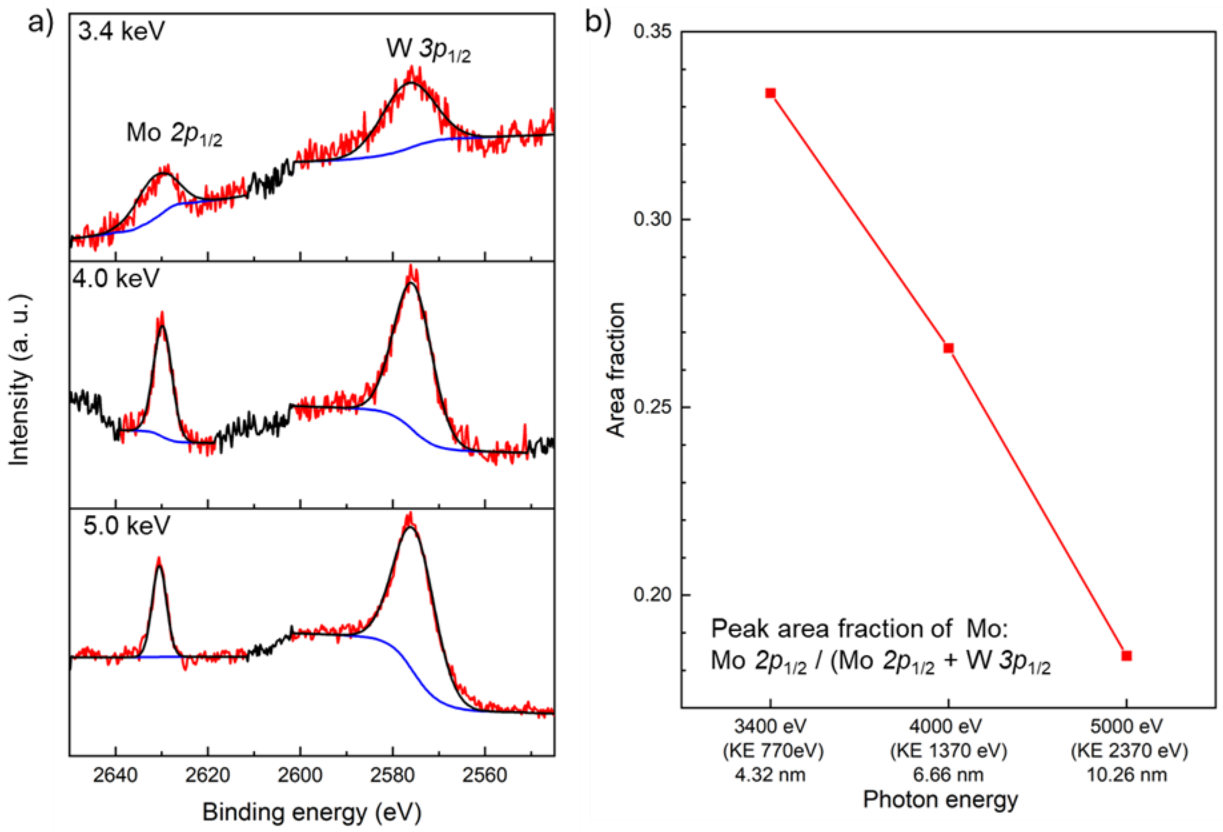

Figure S4: Synchrotron XPS showing the depth-dependent composition profile of Mo in 18% Mo^{6+}:WO_3 with increasing photon energy (PE). (a) Shows the two peaks associated with the Mo $2p_{1/2}$ and W $3p_{1/2}$ at different PEs and the (b) shows the PE-dependent area fraction of the Mo $2p_{1/2}$ peak.

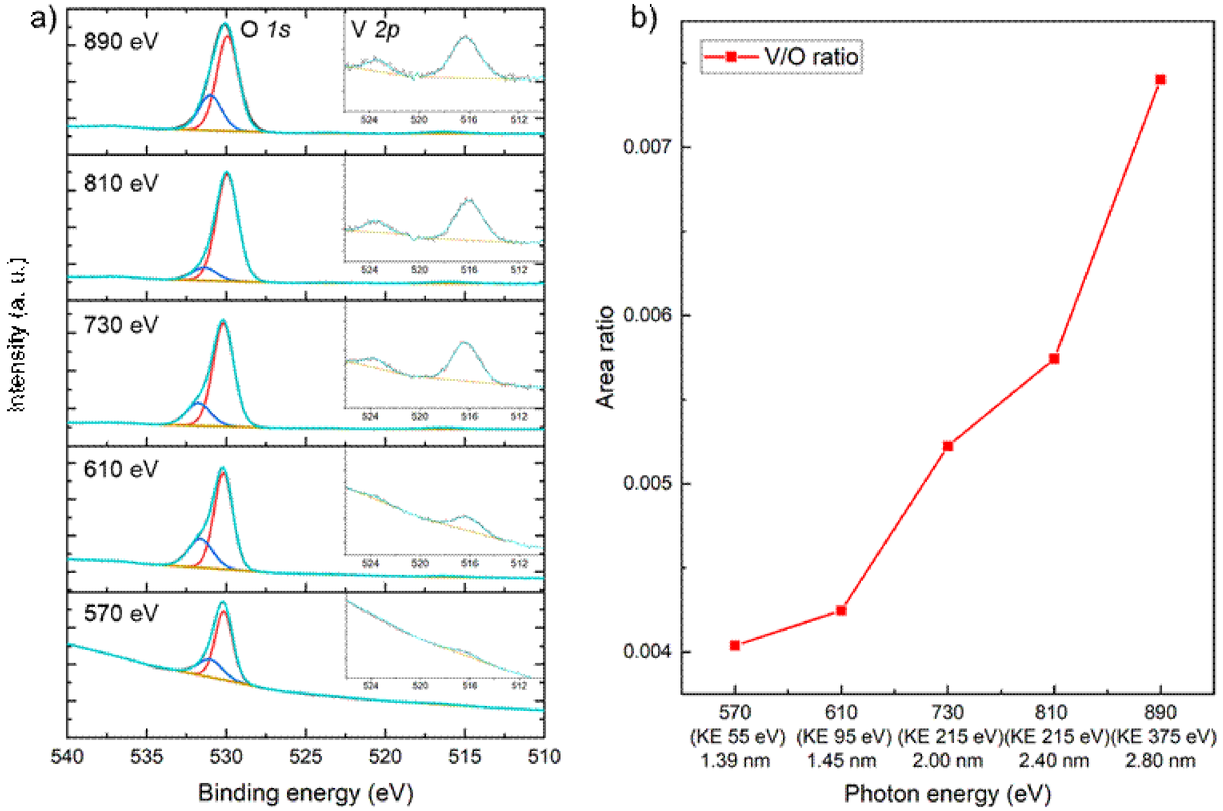

Figure S5: Synchrotron-based XPS analysis of the depth-dependent compositional profile of V in 6% V^{5+}:WO_3 as a function of photon energy (PE). (a) XPS spectra showing the relative intensities of the O *1s* and V *2p* regions at different photon energies; the inset presents a magnified view of the V *2p* region. (b) Photon energy–dependent area ratio of the V $2p_{3/2}$ to O *1s* peaks. The observed variation in the area ratio indicates that the V concentration is lower at the surface compared to the subsurface.

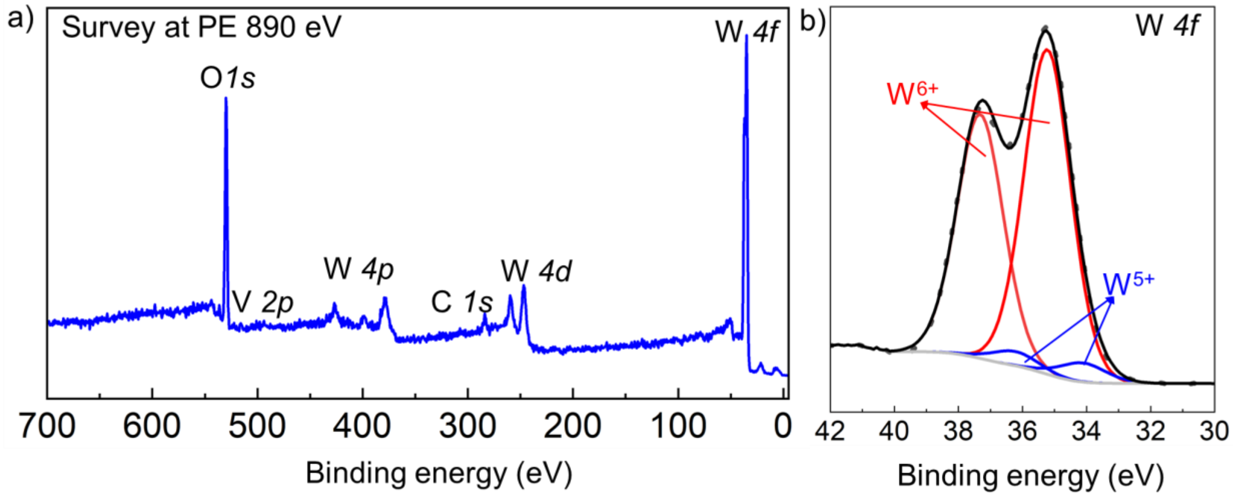

Figure S6. Synchrotron-based XPS survey and W 4f spectra of 6% V^{5+}:WO_3 collected at a photon energy of 890 eV. (a) XPS survey spectrum showing the relative intensities of all elements present in the sample, along with a residual adventitious carbon layer remaining after annealing in 100 mTorr of O_2 at 400 °C. (b) High-resolution W 4f spectrum indicating that tungsten is not entirely in the W^{6+} oxidation state; the presence of W^{5+} features suggests partial oxygen deficiency in the sample.

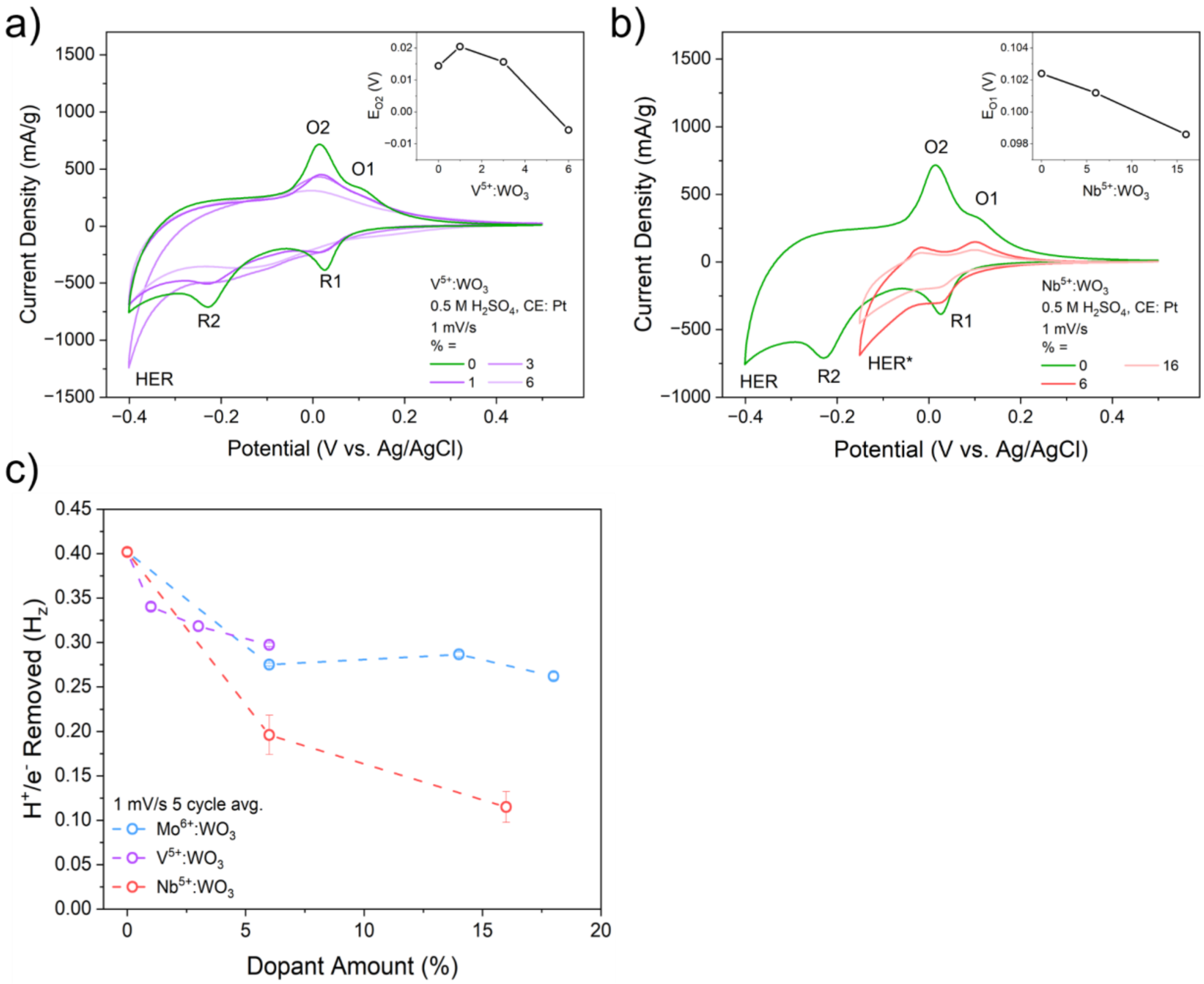

Figure S7: Comparison of CVs collected at 1 mV/s for the aliovalent systems, V^{5+}:WO_3 (a) and Nb^{5+}:WO_3 (b) series, with undoped WO_3. Inset images show the dopant concentration dependent potential shift of the primary oxidation feature (O2 for V^{5+}, O1 for Nb^{5+}). The effect of dopant concentration on the anodic capacity expressed in terms of H^+/e^- removed from the active material during the oxidation sweep for all three dopant-series is shown in (c).

Discussion S2:

Scan Rate Dependent Cyclic Voltammograms (CVs)

Cyclic voltammograms were collected with potential sweep rates of 100, 50, 10, 5, and 1 mV/s to study the kinetic components of PICET for the heteroatom doped samples. For direct comparison, the electrochemical capacities were determined from the anodic potential sweeps to minimize current contributions from parasitic PCET reactions such as hydrogen evolution. The scan rate dependence of the anodic capacity (given in terms of H^+/e^- transferred from the electrode) are shown separately for all three heteroatom doped series with respect to the undoped WO_3 in **Figure S8**. For the three dopant-series, the difference in achieved capacity is more pronounced at slower scan rates, while at the faster scan rates (50 and 100 mV/s) the capacities are comparable to the undoped sample. In some instances (i.e. 6% Mo^{6+} and V^{5+}), the doped materials perform better at faster rates than the undoped sample.

To further assess the differences in rate behavior we conducted b-value analysis of the scan rate dependent CV measurements. The b-value equation is expressed as:

$$i = av^b$$

where i is the peak current magnitude, v is the scan rate, and a and b are fitting parameters, can be used to identify the rate limiting step of an electrochemical ion-coupled electron transfer reaction. The limiting b-values of 0.5 and 1 emerge from semi-infinite diffusion and surface adsorption limitations, respectively. The b-values for all compositions studied here are presented in **Figure S9**. For undoped WO_3, we obtained a b-value of 0.48(3) consistent with a semi-infinite diffusion limited process. All heteroatom doped samples were found to have larger b-values, ranging from 0.52(3) to 0.85(8). These intermediate b-values have been previously attributed to finite diffusion limitations.[10] We suspect that the smaller particle sizes observed for the heteroatom doped samples from SEM and BET measurements contributes to the appearance of finite diffusion limitations. The increases in the b-value can be attributed to changes in the solid-state diffusivity of H^+ or changes in the interfacial PCET reaction kinetics.

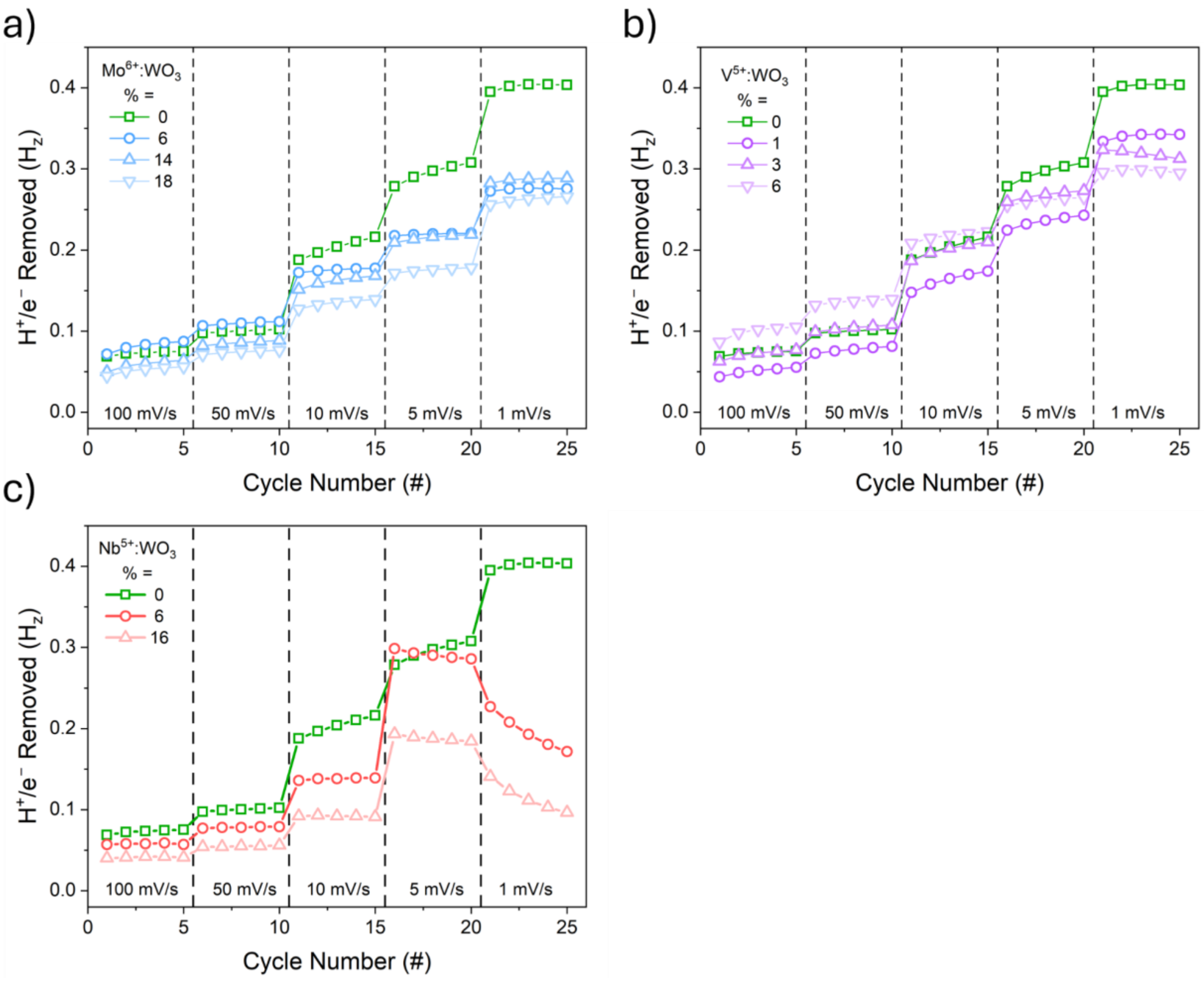

Figure S8: Results of the scan-rate dependent CV experiments for the Mo^{6+}:WO_3 (a), V^{5+}:WO_3 (b), and Nb^{5+}:WO_3 (c) series showing the anodic capacities in terms of H^+/e^- removed from the active material during the oxidation sweep. Potential sweep rates are indicated within their respective figures and cycle number groups.

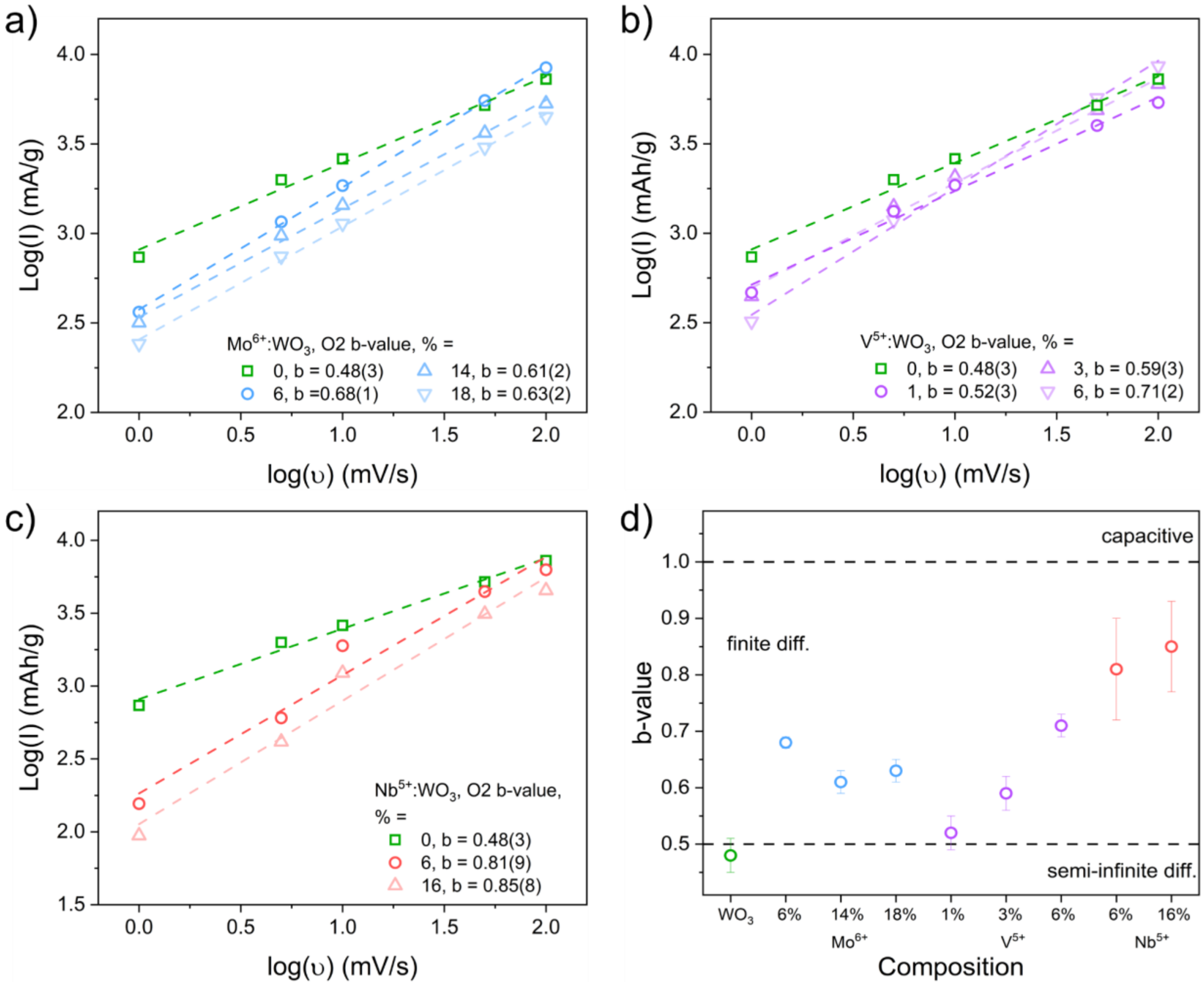

Figure S9: B-value analysis of the O2 redox peak (O1 for Nb-doped) for the Mo-doped (a), V-doped (b), and Nb-doped (c) series taken as the slope of the log-log plot for the scan rate dependence of the peak current. Summary of b-values determined for the three dopant-series (d).

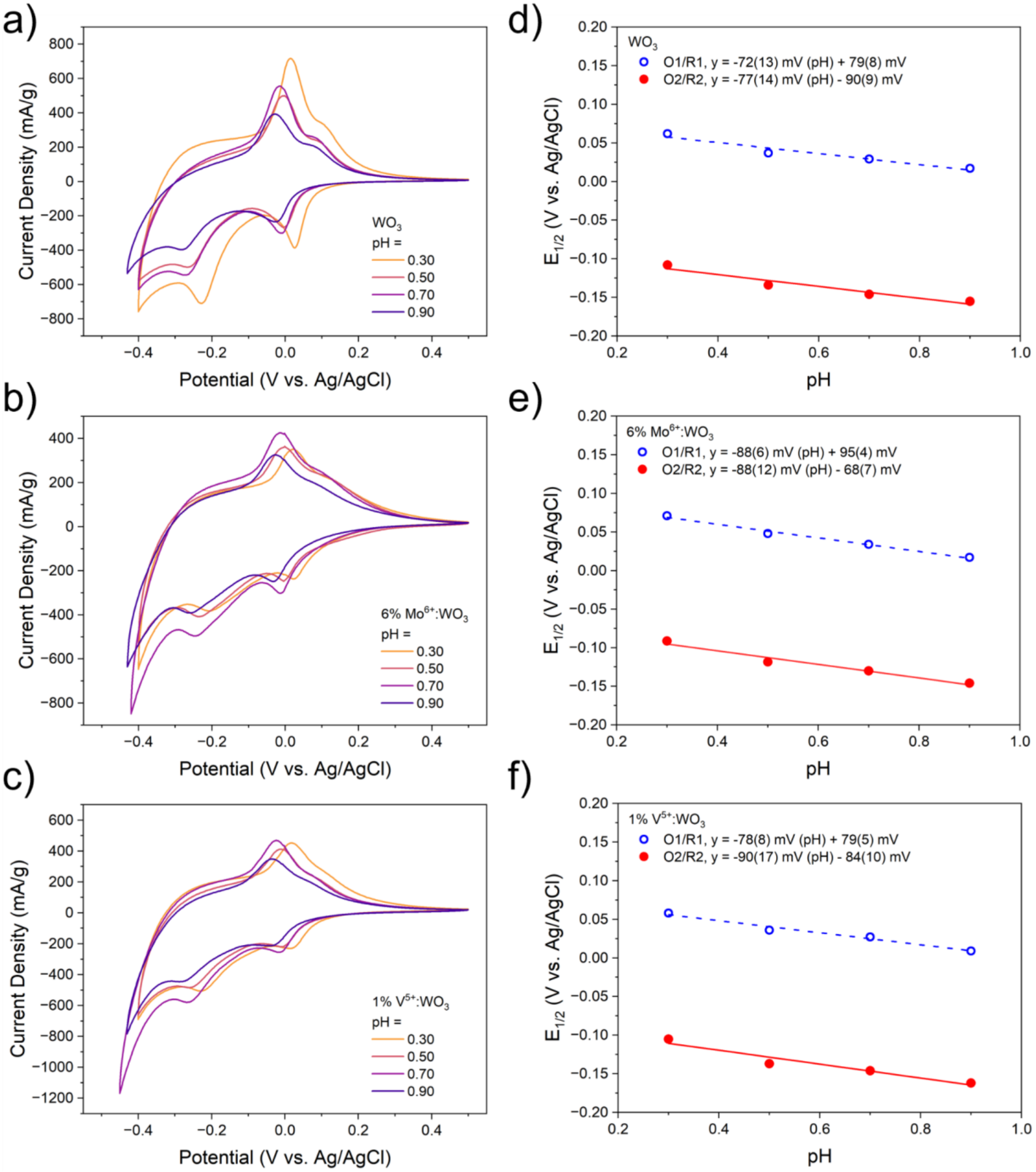

Figure S10: Cyclic voltammograms for pristine WO_3 (a), 6% Mo^{6+}:WO_3 (b), and 1% V^{5+}:WO_3 (c) upon increasing the electrolyte pH. Linear trends for the shifts in the $E_{1/2}$ values of the O1/R1 and O2/R2 redox couples for WO_3 (d), 6% Mo^{6+}:WO_3 (e), and 1% V^{5+}:WO_3 (f).

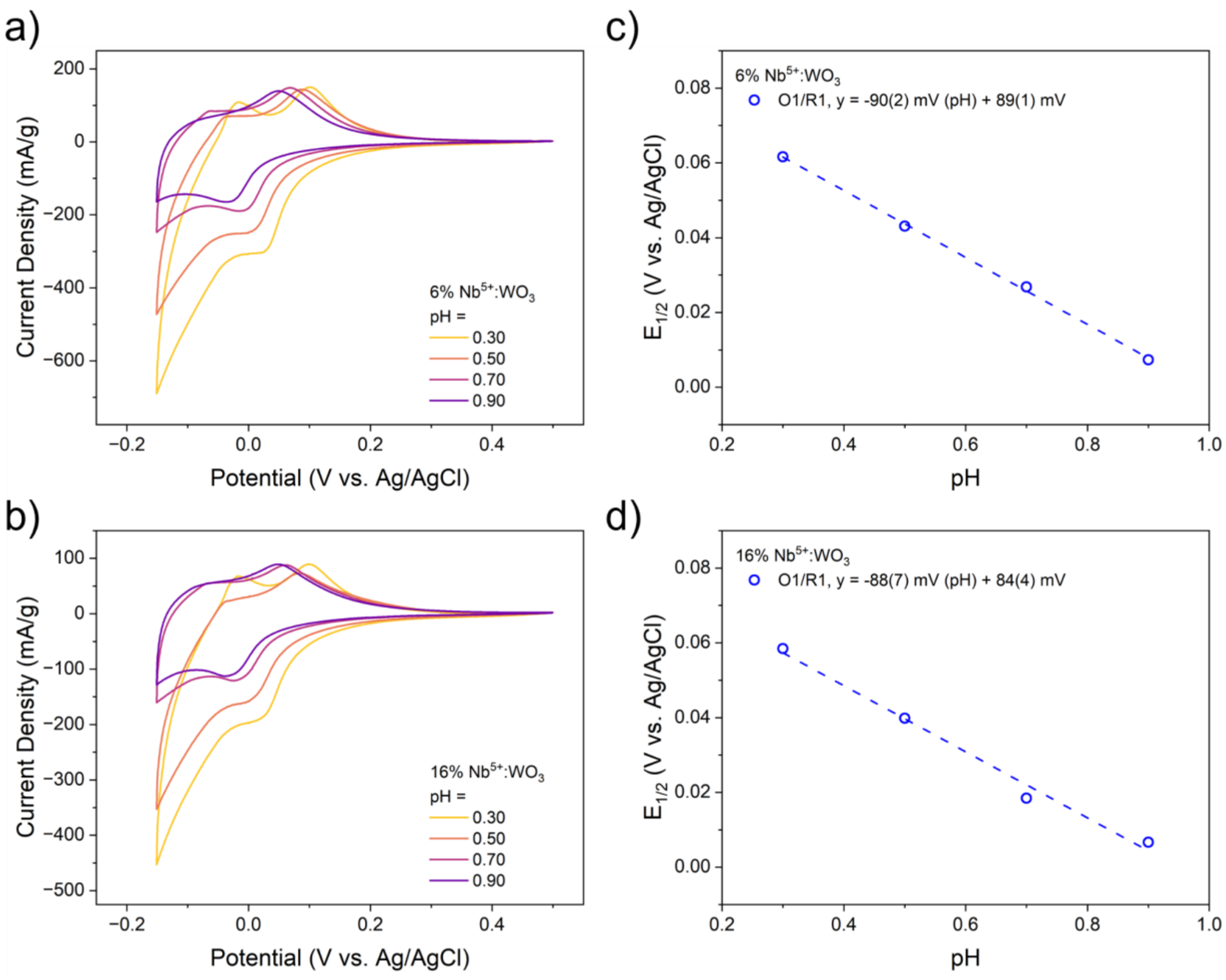

Figure S11: Cyclic voltammograms for 6% Nb^{5+}:WO_3 (a), and 16% Nb^{5+}:WO_3 (b) upon increasing the electrolyte pH. Linear trends for the shifts in the $E_{1/2}$ values of the O1/R1 and O1/R1 redox couples for 6% Nb^{5+}:WO_3 (c), and 16% Nb^{5+}:WO_3 (d).

Equations S1 - S3: Equations used to calculate the pseudocubic (a_p) lattice parameter (Å) for the monoclinic WO_3(S1), tetragonal *P*4/*nmm* H_xWO_3 (S2), and cubic *Pm*-3*m* H_xWO_3 (S3).

Monoclinic (*P*2$_1$/*n*)	$a_p = (a + b + c) / 6$	**Eq. S1**
Tetragonal (*P*4/*nmm*)	$a_p = (2 * (a / \sqrt{(2)}) + c) / 3$	**Eq. S2**
Cubic (*Pm-3m*)	$a_p = a$	**Eq. S3**

Discussion S3:

Operando XRD results for 6%V^{5+}:WO_3

We performed operando SPXRD on 6% V^{5+}:WO_3 in order to compare with the lesser doped 1% V^{5+}:WO_3. The broadened diffraction peaks observed in the lab diffraction measurements (**Fig. 2a**) persist in the synchrotron measurements (**Fig. S12a**) making phase identification difficult. Instead, we chose to model all of the diffraction patterns with the cubic model with the addition of a Gaussian peak strain parameter to account for the change in peak broadness (taken as the full-width at half-maximum - FWHM). We would expect that this strain parameter will decrease as the peaks coalesce. The trend in the cubic lattice parameter follows with the other two doped samples, showing an increase in a_p as the material is reduced. From the strain parameter, we observe a decrease in peak FWHM as the a_p-parameter increases, consistent with the merging of diffraction peaks (**Fig. S12b**). Changes in the strain parameter value are observed upon reduction and oxidation. These regions may be associated with changes in the dominant structural phase present in the sample where the monoclinic cell exists in the regions with the largest strain (multiple partially overlapping peaks) and the cubic structure exists in the region with the lowest strain (single allowed peak). Regions with intermediate strain values may consist of the tetragonal bronze phase or a mixture of bronze phases. Upon reduction, the strain parameter decreases around H_z = 0.10, indicating that the higher symmetry bronze phases are present. Upon oxidation, the strain parameter increases near H_z = 0.25, suggesting oxidation of the bronze phases. As the sample is further oxidized, the strain parameter gradually returns to the initial value. This strain analysis would indicate similar hysteresis in the structural transitions to what was observed for 18% Mo^{6+}:WO_3.

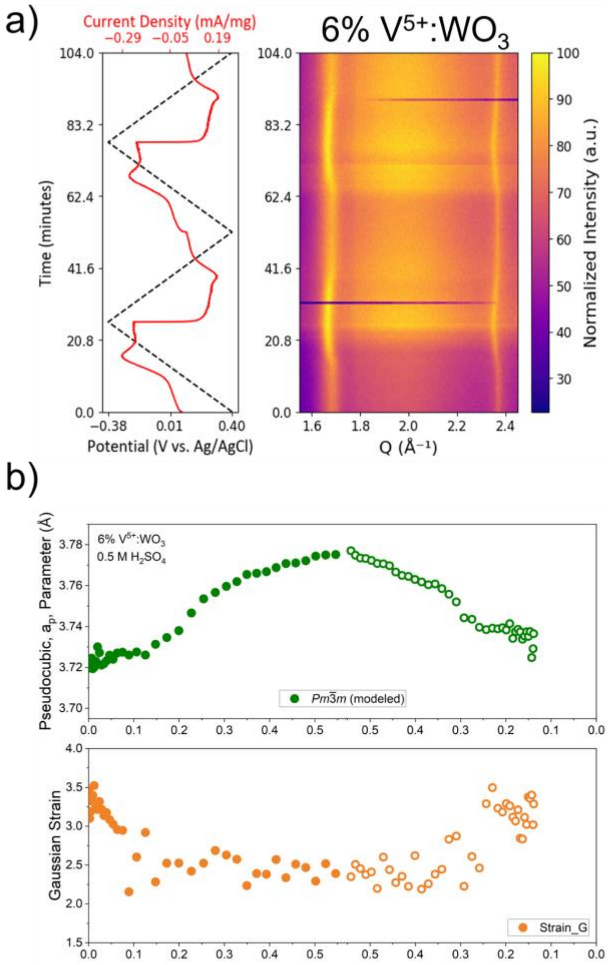

Figure S12: Surface plot obtained from operando SPXRD measurements in 0.5 M H_2SO_4 for and 6% V^{5+}:WO_3 (a). Trend for the Gaussian strain parameter used to model 6% V^{5+}:WO_3 (b). Full circles indicate the reduction sweep, and empty circles indicate the oxidation sweep for the first cycle.

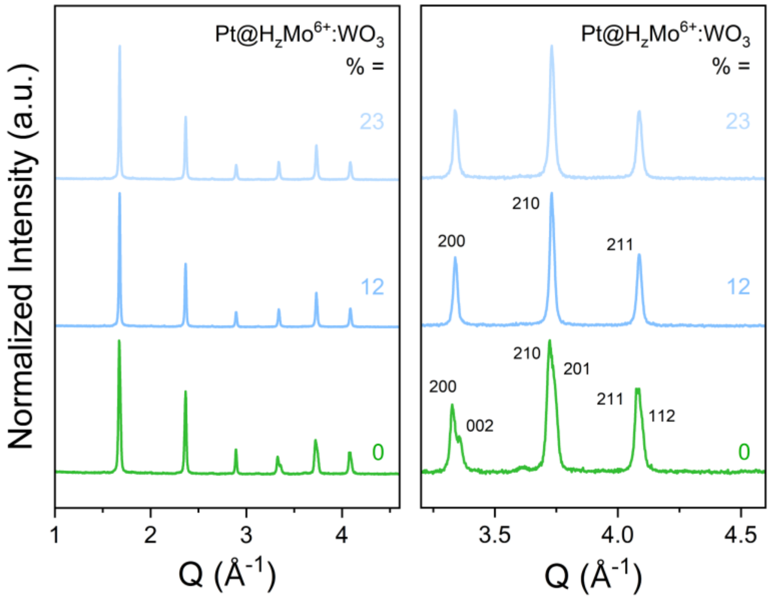

Figure S13: PXRD patterns for the chemically reduced H_zMo^{6+}:WO_3 samples that were sintered at 500°C prior to reduction in order to increase crystallite size and mitigate preferred orientation of the nanoplatelets used for electrochemical analysis.

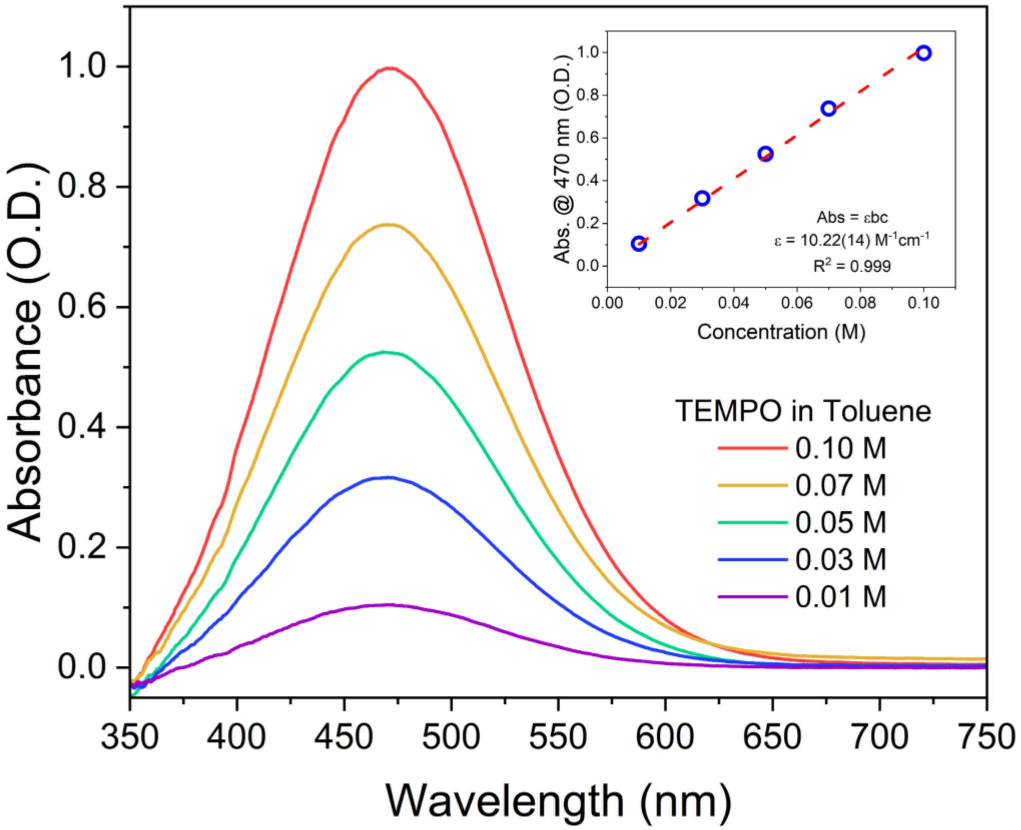

Figure S14: Concentration dependence of the absorbance intensity for TEMPO in toluene. The Beer's law linear trend and extrapolated molar absorption coefficient are given within the inset figure.

a)

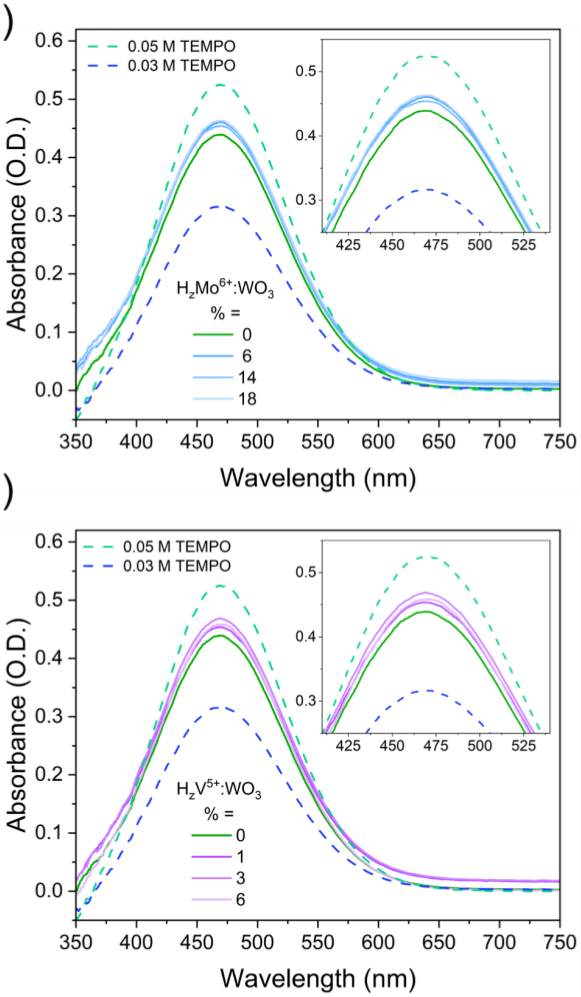

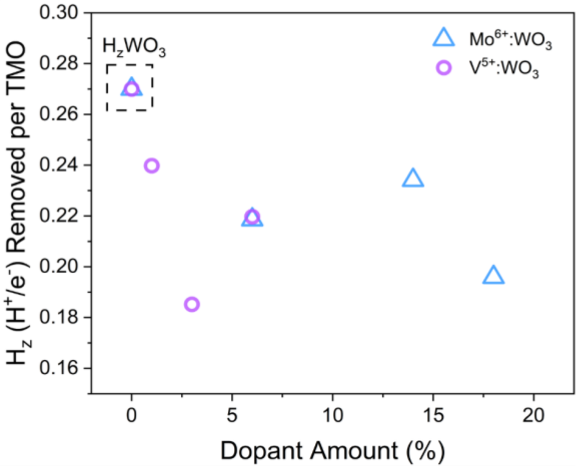

d)

Sample	ΔAbs_{470}	H_z per TMO
WO_3	0.085	0.27
6% $Mo^{6+}:WO_3$	0.064	0.22
14% $Mo^{6+}:WO_3$	0.070	0.23
18% $Mo^{6+}:WO_3$	0.062	0.20
1% $V^{5+}:WO_3$	0.071	0.24
3% $V^{5+}:WO_3$	0.057	0.19
6% $V^{5+}:WO_3$	0.066	0.22

Figure S15: UV-Vis absorbance measurements for TEMPO solutions before and after reaction with the heteroatom doped hydrogen bronzes for the Mo^{6+}-series (a) and V^{5+}-series (b). Summary of hydrogen atom transfer (HAT) results determined from the change in absorbance at 470 nm in graphical form (c) and tabular form (d).

Discussion S4:

Density Functional Theory results:

Three structural arrangements used to evaluate the effect of heteroatom doping on hydrogen binding energies:

1. Without an oxygen vacancy, where the intercalated proton binds to the right oxygen in the atomic sequence (**Fig. S16 left, blue box**).
2. With an oxygen vacancy, where the intercalated proton binds to the middle oxygen (**Fig. S16 left, red box**).
3. With an oxygen vacancy, where the intercalated proton is located between the two metal atoms (**Fig. S16 left, green box**).

The left side of **Fig. S16** includes the most stable configurations for both proton binding sites and oxygen vacancy sites, whereas the right side depicts less stable configurations. Additional oxygen vacancy and hydrogen binding sites have also been considered, but they become progressively less exothermic with increasing distance from the dopants.

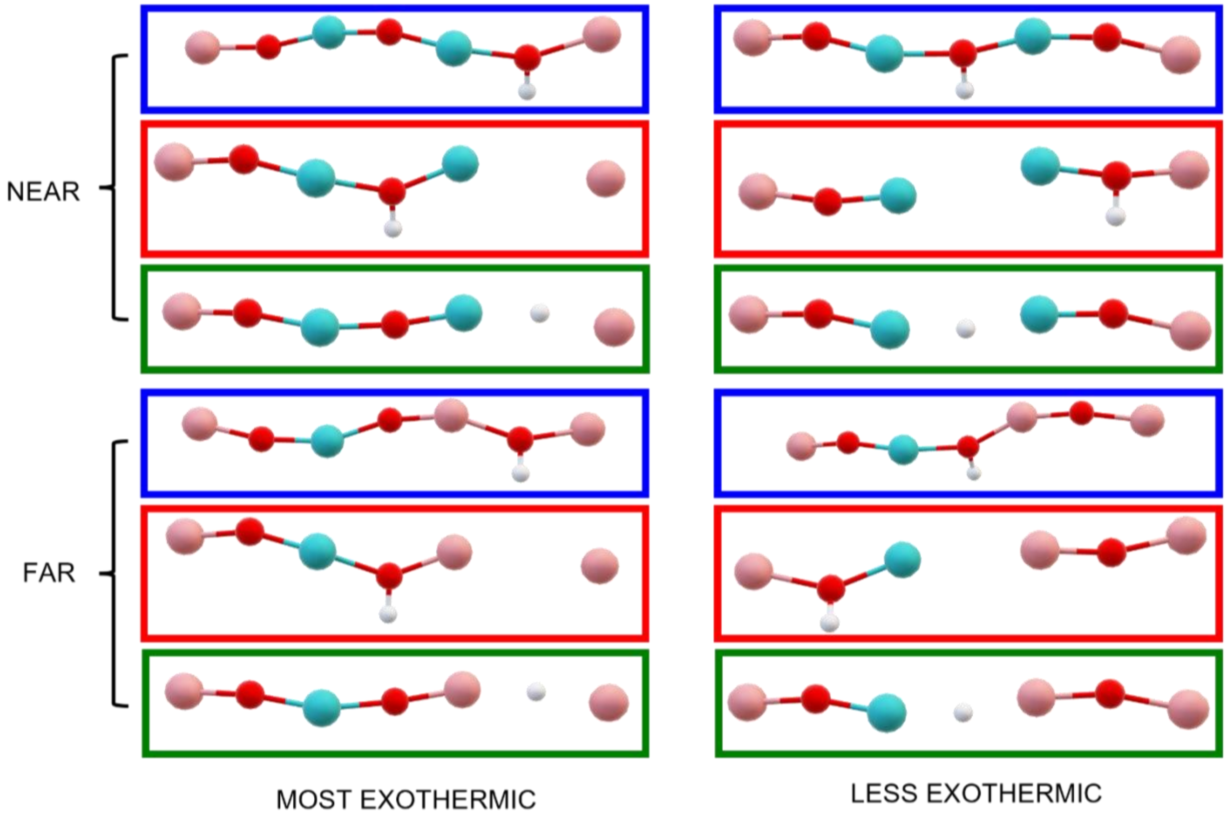

Figure S16: Sequence of atoms of interest. Cyan: W, Mo, Nb, or V; red: O; pink: W; white: H. In blue contour: No O vacancy, H bound to right O. In red contour: O vacancy, H bound to middle O. In green contour: O vacancy, H positioned between two metals. The top three sequences represent the near configurations, while the bottom three represent the far ones.

Table S2: Net Bader charges, hydrogen binding energies (HBE), and oxygen vacancy formation energies (OVFE) for the investigated structures. The dashed line separates structures without vacancies (shown above the line) from those with oxygen vacancies (denoted as X, shown below the line). The oxygen atoms (O) in red represent the binding sites of the inserted hydrogen atoms (HBE on Oxygen).

	Net Charge								HBE on Oxygen [eV]	HBE between metals [eV]	OVFE [eV]
	D	O	M	O/X	W	O	W	H			
Mo-O-Mo-**O**-W-O-W	2.67	-0.93	2.69	-0.99	2.99	-0.93	2.97	0.59	-0.90		
V-O-V-**O**-W-O-W	2.17	-0.81	2.14	-0.94	2.98	-0.91	2.99	0.60	-3.01		
Nb-O-Nb-**O**-W-O-W	2.69	-1.01	2.66	-1.02	2.99	-1.00	2.99	0.62	-2.68		
Mo-O-W-**O**-W-O-W	2.68	-0.99	3.00	-0.99	2.98	-0.99	2.97	0.63	-0.74		
V-O-W-**O**-W-O-W	2.17	-0.95	3.00	-0.95	2.97	-0.95	2.99	0.60	-2.68		
Nb-O-W-**O**-W-O-W	2.66	-1.02	3.01	-1.03	2.99	-1.01	3.00	0.59	-2.90		
W-O-W-**O**-W-O-W	2.94	-1.02	2.99	-1.02	2.86	-1.01	2.95	0.75	-0.17		
Mo-**O**-Mo-X-W-O-W	2.49	-0.88	2.56		2.89	-0.99	2.96	0.61	0.14	-0.54	0.71
V-**O**-V-X-W-O-W	2.12	-0.85	2.16		2.92	-0.98	2.97	0.60	-0.30	1.58	-3.51
Nb-**O**-Nb-X-W-O-W	2.58	-0.99	2.67		2.90	-0.99	2.99	0.61	-0.12	1.15	-2.94
Mo-**O**-W-X-W-O-W	2.86	-0.95	2.59		2.89	-0.97	2.97	0.60	0.47	-0.73	1.03
V-**O**-W-X-W-O-W	2.9	-0.93	2.17		2.91	-0.96	2.99	0.65	-0.27	0.92	-2.69
Nb-**O**-W-X-W-O-W	2.9	-0.98	2.66		2.91	-1.02	3.00	0.66	-0.28	0.75	-2.48
W-**O**-W-X-W-O-W	2.78	-0.99	2.95		2.74	-0.98	2.94	0.75	0.24	-1.53	2.09

The net charges of the atoms of interest were calculated for the non-protonated structures (except for the case of hydrogen). This analysis shows no significant charge difference between the rightmost oxygen (~ –1) and the rightmost tungsten (~ +3). However, the two metal atoms that bind with the oxygen which creates the vacancy (3rd and 5th column) display distinct behavior: in Mo-doped (isovalent) and pristine structures, their charges decrease upon oxygen vacancy formation, while in Nb- and V-doped (aliovalent) structures, they remain nearly unchanged. The same pattern is observed for the left metal (1st column). A slight reduction in the hydrogen atom charge is also observed in doped structures compared to the pristine case. Beyond these trends, no clear correlation between atomic charges and HBEs is found.

References:

(1) Shannon, R. D. *Revised Effective Ionic Radii and Systematic Studies of Interatomie Distances in Halides and Chaleogenides*; 1976; Vol. 32.

(2) Salje, E.; Gehlig, R.; Viswanathan, K. Structural Phase Transition in Mixed Crystals $W_xMo_{1-x}O_3$. *J. Solid State Chem.* **1978**, *25* (3), 239–250. https://doi.org/10.1016/0022-4596(78)90109-3.

(3) Roth, R. S.; Wadsley, A. D. Multiple Phase Formation in the Binary System Nb_2O_5–WO_3. I. Preparation and Identification of Phases . *Acta Crystallogr.* **1965**, *19* (1), 26–32. https://doi.org/10.1107/s0365110x65002712.

(4) Yu, Z.; Yu, D.; Wang, X.; Huang, M.; Hou, Y.; Lin, W.; Anpo, M.; Yu, J. C.; Zhang, J.; Wang, X. Photoinduced Formation of Oxygen Vacancies on Mo-Incorporated WO_3 for Direct Oxidation of Benzene to Phenol by Air. *J. Am. Chem. Soc.* **2025**, *147* (16), 13885–13892. https://doi.org/10.1021/jacs.5c02086.

(5) Li, H.; Abdelgaid, M.; Paudel, J. R.; Holzapfel, N. P.; Augustyn, V.; Mckone, J. R.; Mpourmpakis, G.; Crumlin, E. J. Unveiling of Hydrogen Spillover Mechanisms on Tungsten Oxide Surfaces. *J. Am. Chem. Soc.* **2025**, *147*, 6472–6479. https://doi.org/10.1021/jacs.4c13711.

(6) Najbar, M.; Camra, J.; Bialas, A.; Weselucha-Birczynska, A.; Borzecka-Prokop, B.; Delevoye, L.; Klinowski, J. Structural Studies of V_2O_5–WO_3 and WO_3–V_2O_5 Solid Solutions. *Phys. Chem. Chem. Phys.* **1999**, *1*, 4645–4648.

(7) Inglot, A.; Najbar, M.; Borzęcka-Prokop, B. Phases in Vanadium Pentoxide-Tungsten Trioxide Catalysts. *J. Chem. Soc. Faraday Trans.* **1995**, *91* (1), 145–148. https://doi.org/10.1039/FT9959100145.

(8) Mitchell, J. B.; Lo, W. C.; Genc, A.; Lebeau, J.; Augustyn, V. Transition from Battery to Pseudocapacitor Behavior via Structural Water in Tungsten Oxide. *Chem. Mater.* **2017**, *29* (9), 3928–3937. https://doi.org/10.1021/acs.chemmater.6b05485.

(9) Spencer, M. A.; Fortunato, J.; Augustyn, V. Electrochemical Proton Insertion Modulates the Hydrogen Evolution Reaction on Tungsten Oxides. *J. Chem. Phys.* **2022**, *156* (6). https://doi.org/10.1063/5.0082459.

(10) Chagnot, M.; Abello, S.; Dawlaty, J.; Rodriguez-Lopez, J.; Zhang, C.; Augustyn, V. Influence of Finite Diffusion on Cation Insertion- Coupled Electron Transfer Kinetics in Thin Film Electrodes. *J. Electrochem. Soc.* **2024**, *171*, 010527. https://doi.org/10.1149/1945-7111/ad1d98.
